# Mitochondrial DNA-driven intercellular communication networks in post-infarction ventricular remodeling: the three-threshold model of cGAS-STING activation

**DOI:** 10.3389/fimmu.2026.1852079

**Published:** 2026-05-25

**Authors:** Chao Meng, Xiao Xia, Yiying Liu, Jun Li, Shiyi Tao, Xuanchun Huang, Yonghao Li

**Affiliations:** Department of Cardiology, Guang’anmen Hospital, China Academy of Chinese Medical Sciences, Beijing, China

**Keywords:** cGAS-STING pathway, mitochondrial DNA, myocardial infarction, three-threshold model, ventricular remodeling

## Abstract

Mitochondrial DNA (mtDNA) has long been recognized as an intracellular damage−associated molecular pattern, but emerging evidence reveals its role as an intercellular messenger driving post−infarction ventricular remodeling. This review systematically elaborates the transition of mtDNA from an “intracellular DAMP” to an “intercellular messenger” and proposes a conceptual framework termed the “three−threshold model”, which integrates existing evidence but requires direct experimental validation. The three thresholds are defined as the release threshold (mitophagy−controlled mtDNA leakage), the transmission threshold (efficiency of intercellular transfer), and the activation threshold (sensitivity of STING signaling). mtDNA is transferred between cells via four modes—naked mtDNA, Ambra1^+^ sEVs, mt−sEVs, and intact mitochondria—mediating inflammation, fibrosis, and vascular dysfunction, respectively. These pathological effects continuously lower all three thresholds through positive feedback loops, driving irreversible remodeling. Time−window−based combination strategies offer a new paradigm for precision intervention. This framework integrates the entire process from mtDNA generation to intercellular transfer and downstream signaling, providing a systematic basis for precision intervention in post−infarction remodeling. Importantly, several components of this model are based on preliminary or indirect evidence and await independent confirmation.

## Graphical Abstract

**Figure:**
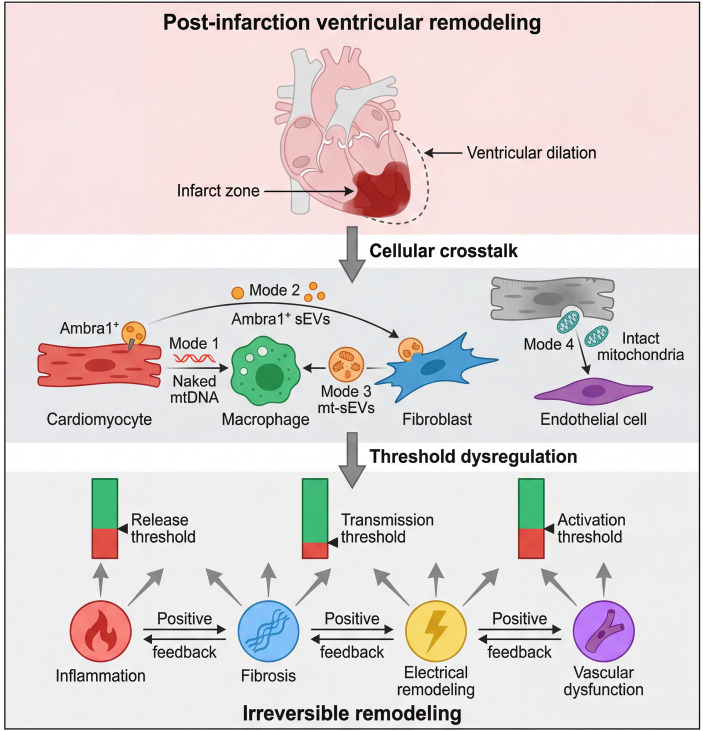
This three−layer diagram illustrates the progression from organ−level pathology to cellular crosstalk and molecular threshold dysregulation in post−infarction ventricular remodeling. Upper layer: Infarcted and dilated heart. Middle layer: Four cell types (cardiomyocyte, macrophage, fibroblast, endothelial cell) and four modes of intercellular mtDNA transfer (naked mtDNA, Ambra1^+^ sEVs, mt−sEVs, intact mitochondria). Lower layer: The three thresholds (release, transmission, activation) are progressively lowered by positive feedback loops involving inflammation, fibrosis, electrical remodeling, and vascular dysfunction, leading to irreversible remodeling.

## 1 Introduction

Post-infarction ventricular remodeling is a major pathological driver of heart failure after acute myocardial infarction (AMI). Although reperfusion therapy has significantly reduced acute mortality, 25–30% of patients still develop progressive ventricular remodeling and eventually heart failure (1). Ventricular remodeling involves multiple processes, including cardiomyocyte death, inflammatory infiltration, fibroblast activation, extracellular matrix deposition, and vascular remodeling. These events do not occur in isolation but are coordinated through a complex “social network” of intercellular crosstalk among various cell types. Understanding the mechanisms underlying this network is essential for developing novel strategies to prevent post-infarction heart failure.

In recent years, the cGAS (cyclic GMP-AMP synthase)-STING (stimulator of interferon genes) pathway has been identified as a key signaling axis driving sterile inflammation and ventricular remodeling after myocardial infarction. Traditionally, mitochondrial DNA (mtDNA) has been viewed as a damage-associated molecular pattern (DAMP) that activates the intracellular cGAS-STING pathway, mediating sterile inflammation. cGAS recognizes cytosolic double-stranded DNA and catalyzes the synthesis of cGAMP (cyclic GMP-AMP), which then binds STING and induces its translocation from the endoplasmic reticulum (ER) to the Golgi apparatus. This can lead to the phosphorylation of IRF3 (interferon regulatory factor 3) and NF-κB (nuclear factor kappa-light-chain-enhancer of activated B cells) via TBK1 (TANK-binding kinase 1), thereby contributing to the initiation of the transcription of type I interferons and pro-inflammatory cytokines (2, 3). However, this framework cannot explain why distant cells mount strong inflammatory or fibrotic responses even when mtDNA leakage is limited.

Breakthrough studies have shown that mtDNA can be actively packaged and transferred to neighboring cells, serving as an intercellular “messenger.” Four modes of transfer have been identified so far: Mode 1, stressed cardiomyocytes release naked mtDNA, which is engulfed by macrophages, activating the cGAS-STING pathway and forming a positive feedback loop in cardiomyocyte–macrophage crosstalk (4); Mode 2, cardiomyocytes secrete Ambra1^+^ small extracellular vesicles (sEVs) carrying mtDNA, which are taken up by fibroblasts, leading to cGAS-STING activation and direct promotion of fibrosis (5); Mode 3, activated fibroblasts release mt-sEVs (mitochondria-enriched small extracellular vesicles) that are internalized by macrophages, amplifying the inflammatory response (6); Mode 4, under severe injury, intact mitochondria are released and activate endothelial cells via the IFI16 (interferon gamma-inducible protein 16)-STING axis (7). Together, these findings indicate that mtDNA is not merely a passive byproduct of cell injury but an active signal orchestrating multicellular responses.

Based on this conceptual shift, the present review puts forward a hypothetical framework – the “three-threshold model” – that conceptually divides the overactivation of the mtDNA-cGAS-STING axis into three sequential and interconnected regulatory nodes: the release threshold (the critical point at which mitophagy controls mtDNA leakage from mitochondria into the cytosol, determined by the PINK1/Parkin pathway, autophagic flux integrity, and mtDNA oxidative modification); the transmission threshold (the efficiency of mtDNA transport across cells via vesicles or other carriers, depending on extracellular stability, carrier form, uptake efficiency, and endosomal escape capacity); and the activation threshold (the sensitivity of the STING pathway in recipient cells, which exhibits marked cell type specificity, being lowest in macrophages and highest in fibroblasts). This conceptual integration synthesizes the entire process from mtDNA generation to intercellular transfer and downstream signaling, and provides a testable framework for understanding the threshold-based regulatory network underlying multicellular crosstalk in post-infarction remodeling. It should be emphasized that many elements of this model, particularly the quantitative definitions of each threshold and the detailed mechanisms of transmission threshold regulation, remain to be directly validated by future studies.

## 2 Setting the release threshold: mitophagy defects and mtDNA leakage

### 2.1 Molecular mechanisms and genetic evidence of mitophagy

Mitophagy is a key quality control mechanism that selectively eliminates damaged mitochondria. Its molecular mechanisms are mainly divided into the ubiquitin-dependent pathway (mediated by PINK1/Parkin) and the receptor-mediated pathway (mediated by FUNDC1, BNIP3/NIX) (8, 9). These two pathways play complementary and spatiotemporally specific roles in myocardial ischemia-reperfusion injury, and their integrity is essential for maintaining low-level mtDNA release and preventing sterile inflammation.

The PINK1/Parkin pathway is the most intensively studied mitophagy mechanism. PINK1 (PTEN-induced putative kinase 1) acts as a sensor of mitochondrial membrane potential. In healthy mitochondria, PINK1 is imported into the inner membrane in a membrane-potential-dependent manner and rapidly degraded. When mitochondria are damaged and the membrane potential is lost, PINK1 stabilizes on the outer mitochondrial membrane, recruits and phosphorylates Parkin (an E3 ubiquitin ligase), initiating ubiquitination of mitochondrial proteins and forming autophagy recognition signals, ultimately leading to the clearance of damaged mitochondria (1, 10). Studies have shown that the PINK1/Parkin pathway plays a protective role in pressure overload-induced cardiac remodeling: PINK1 deficiency increases mtDNA leakage and hyperactivates the cGAS-STING pathway, aggravating cardiac hypertrophy; conversely, PINK1 overexpression suppresses the pathway and attenuates remodeling (1, 11) [Strong evidence]. Furthermore, genetic manipulation of Parkin has confirmed the central role of this axis: Parkin silencing exacerbates mitochondrial fragmentation and mtDNA release, whereas cardiomyocyte-specific Parkin overexpression reduces cytosolic mtDNA accumulation, inhibits STING activation, and alleviates ischemia-reperfusion injury (11, 12).

The receptor-mediated pathway does not depend on ubiquitination. Instead, autophagy receptors on the outer mitochondrial membrane directly interact with LC3 (microtubule-associated protein 1 light chain 3). FUNDC1 (FUN14 domain-containing protein 1) is a key receptor for mitophagy under hypoxic conditions; its N-terminal LC3-interacting region is activated by dephosphorylation induced by hypoxia (13). BNIP3 (BCL2/adenovirus E1B 19 kDa protein-interacting protein 3) and its homolog NIX (also known as BNIP3L) are another important class of mitophagy receptors, upregulated under hypoxia and mediating selective mitochondrial clearance (8). In an acute hypoxia model, GSK-3α (glycogen synthase kinase-3α) can promote BNIP3 expression by upregulating HIF-1α (hypoxia-inducible factor-1α) and FOXO3a (forkhead box O3a), inducing mitophagy independently of the PINK1/Parkin pathway (14). This mechanism has been validated in human cardiomyocytes, but its persistence in chronic post-infarction remodeling remains unclear.

The two pathways exhibit spatiotemporal functional specialization: during the acute ischemic phase, the receptor-mediated pathway (FUNDC1 and BNIP3) responds rapidly; during the chronic remodeling phase, the PINK1/Parkin pathway exerts more sustained mitochondrial quality control (1, 11, 13, 14). There may be cross-regulation between the two pathways, jointly maintaining mitochondrial homeostasis (8).

The above genetic evidence has also been validated in immune cells: in aged macrophages, decreased PINK1/Parkin-mediated mitochondrial ubiquitination together with impaired lysosomal function (via the mTOR/TFEB signaling pathway) leads to defective mitophagic flux, making mtDNA more prone to cytosolic leakage and subsequent cGAS-STING activation (15). (Note: mTOR = mechanistic target of rapamycin; TFEB = transcription factor EB).

In summary, the PINK1/Parkin and receptor-mediated pathways together constitute the molecular basis of mitophagy in cardiomyocytes. Genetic evidence supports the concept of a causal chain linking mitophagy defects to mtDNA leakage and cGAS-STING activation. This axis is conserved in both cardiomyocytes and macrophages, providing a theoretical foundation for strategies aimed at enhancing mitophagy [Strong evidence].

### 2.2 Autophagic flux integrity: a quality control checkpoint beyond initiation

The effectiveness of mitophagy depends not only on the activation of autophagy receptors (initiation) but also on the efficiency of autophagosome-lysosome fusion and cargo degradation—i.e., the integrity of autophagic flux. When autophagic flux is blocked, even enhanced autophagy initiation fails to effectively degrade damaged mitochondria, leading to abnormal accumulation of mtDNA in the cytosol and subsequent activation of the cGAS-STING pathway, which means a lowered release threshold (15, 16).

In myocardial infarction models, impaired autophagic flux (manifested as autophagosome accumulation and impaired autophagolysosome formation) has been shown to correlate with adverse remodeling (17). Non-pathological chemical stimuli (e.g., silica nanoparticles) also confirm that blockade of autophagic flux leads to mtDNA leakage and cGAS-STING activation (16), although the physiological relevance of these findings awaits further validation.

Comorbidities such as aging and diabetes lower the release threshold by damaging autophagic flux integrity, which may explain why these patients experience more severe post-infarction remodeling. In aged macrophages, decreased PINK1/Parkin-mediated mitochondrial ubiquitination together with impaired lysosomal function (via the mTOR/TFEB signaling pathway) leads to defective autophagic flux, making mtDNA more prone to cytosolic leakage and subsequent cGAS-STING activation (15). In a type 2 diabetes mellitus model of myocardial infarction, diabetic mice showed markedly impaired autophagic flux in the peri-infarct region, accompanied by increased mtDNA release, hyperactivation of the AIM2 (absent in melanoma 2) and NLRC4 (NLR family CARD domain-containing protein 4) inflammasomes, and exacerbated cardiomyocyte death (18).

In summary, autophagic flux integrity is a key determinant of a high release threshold. Impairment of autophagic flux—whether due to lysosomal dysfunction, aging, or metabolic stress—can independently of autophagy initiation defects lead to mtDNA accumulation and cGAS-STING hyperactivation, i.e., a lowered release threshold [Moderate evidence].

### 2.3 mtDNA oxidative modification: enhanced immunogenicity and accelerated leakage

In addition to mitophagy defects, oxidative modification of mtDNA represents another independent pathway that lowers the release threshold, and the two mechanisms can synergistically exacerbate mtDNA leakage. Mitochondria are the primary source of intracellular reactive oxygen species (ROS). Under pathological conditions such as ischemia-reperfusion, diabetes, and aging, excessive mitochondrial ROS production leads to oxidative stress (19, 20). mtDNA is highly susceptible to oxidative damage because it lacks histone protection and has limited DNA repair capacity. Oxidatively modified mtDNA (with 8-hydroxy-2′-deoxyguanosine, 8-OHdG, as a major marker) not only exhibits enhanced immunogenicity but also directly promotes mtDNA leakage by damaging the mitochondrial membrane (e.g., by inducing opening of the mitochondrial permeability transition pore, mPTP) (19).

Total DNA oxidative damage after myocardial infarction has been widely reported (20–22). However, direct evidence for mtDNA-specific oxidative modification comes mainly from models of diabetic and chronic kidney disease. In diabetic models, high glucose/palmitate stimulation triggers a burst of mitochondrial ROS, leading to mtDNA oxidation and cytosolic leakage, which in turn activates cGAS-STING, inducing cardiomyocyte pyroptosis, inflammation, and remodeling (4, 19). In a chronic kidney disease-associated cardiac remodeling model, myocardial mitochondrial oxidative damage was identified as an early event in cardiac remodeling: oxidatively damaged mtDNA released into the cytosol activates the STING-NF-κB pathway, thereby driving cardiac hypertrophy (23). Although extrapolation of these findings to pure myocardial infarction models requires caution, ischemia-reperfusion itself induces strong oxidative stress, and mtDNA oxidative modification is likely to play a similar role.

Based on the above evidence, mtDNA oxidative modification may lower the release threshold through two pathways. On the one hand, oxidative damage increases mitochondrial membrane permeability, promoting passive mtDNA leakage. On the other hand, preliminary studies suggest that oxidative modification may enhance the binding affinity of mtDNA for cGAS (19), although this mechanism awaits direct validation. The level of oxidative stress is a key intrinsic determinant of the release threshold, and together with mitophagy defects constitutes the two major regulatory pillars of the release threshold [Moderate evidence].

It should be emphasized that in severe myocardial infarction, extensive cardiomyocyte necrosis is a dominant form of cell death during acute ischemia/reperfusion (24, 25). Necrotic cells become leaky and release a subset of damage−associated molecular patterns (DAMPs), including large quantities of mtDNA, which may overwhelm the modulatory effects of oxidative modification on the release threshold (24, 26). The concept that oxidative modification lowers the release threshold applies primarily to sublethal stress or mitochondrial leakage in surviving cells, rather than to acute necrotic cell death (26, 27). In contrast, during the subacute and chronic phases, oxidative modification and mitophagy defects likely play more prominent roles in regulating mtDNA leakage from viable but stressed cells (25). Furthermore, the enhanced binding affinity of oxidized mtDNA for cGAS awaits direct validation (see above). Therefore, the relative contribution of oxidative modification to mtDNA leakage should be carefully considered across different post−infarction phases (acute necrosis vs. subacute/chronic remodeling).

### 2.4 Release threshold model: conceptual integration and cross-organ validation

Based on the discussions in Sections 2.1–2.3, this section proposes the concept of the “release threshold” as a putative first checkpoint of mtDNA-cGAS-STING activation. Currently, the quantitative relationship between mitophagy efficiency, oxidative modification, and the release threshold remains undefined; this concept serves as an integrative hypothesis awaiting rigorous testing. The release threshold is conceptually defined as the critical point at which mtDNA escapes from mitochondria into the cytosol, and its height is postulated to be determined by mitophagy efficiency, the degree of mtDNA oxidation, and the mitochondrial damage load (i.e., the number of damaged mitochondria exceeding the clearance capacity of mitophagy). According to the level of the release threshold, three pathophysiological states can be distinguished: physiological homeostasis (high threshold, minimal mtDNA release); compensation (moderate threshold, low-level mtDNA release, possibly representing a reversible compensatory reaction, although its precise protective role remains to be validated); and decompensation (low threshold, massive mtDNA release with excessive cGAS-STING activation, observed in myocardial infarction, diabetic cardiomyopathy, etc.).

This causal chain has also been observed in acute ischemia-reperfusion injury in organs such as the liver, kidney, and intestine (28–31), suggesting that the release threshold mechanism may be conserved across organs. The release threshold is proposed as the first gateway of the “three-threshold model”: it determines whether mtDNA can leak from mitochondria (the “source”), and together with the “activation threshold” (the “terminal”, determining whether recipient cells respond) and the “transmission threshold” (the “bridge”, determining whether mtDNA reaches recipient cells) described in Chapters 3 and 4, respectively, constitutes a stepwise and fully interconnected regulatory network. The exact quantitative values of the release threshold (e.g., mtDNA concentration or flux) and the proposed three-state classification remain speculative at present; these concepts serve as an integrative hypothesis awaiting rigorous experimental testing.

### 2.5 Mechanisms of mtDNA release from donor cells

The release of mtDNA from donor cells into the extracellular space is a prerequisite for intercellular transfer. This process involves two conceptually distinct steps: the escape of mtDNA from the mitochondrial matrix into the cytosol, followed by its export across the plasma membrane into the extracellular milieu. The former has been relatively well studied, whereas the latter remains incompletely understood.

The passage of mtDNA from mitochondria into the cytosol is primarily mediated by inner and outer membrane permeabilization events. A major pathway involves opening of the mitochondrial permeability transition pore (mPTP), a non-selective inner membrane channel that allows passive leakage of matrix contents, including mtDNA, when triggered by calcium overload or oxidative stress (32, 33). The molecular identity, structure, and functional regulation of the mPTP have long been debated; recent consensus suggests that it is composed of F1Fo ATP synthase, adenine nucleotide translocase (ANT), and cyclophilin D (32). In myocardial ischemia-reperfusion (I/R) injury, mPTP opening is a well-established hallmark of mitochondrial dysfunction, and its pharmacological inhibition effectively reduces mtDNA release and subsequent cGAS-STING activation (20). A second, mechanistically distinct pathway involves BAX/BAK-mediated mitochondrial outer membrane permeabilization (MOMP). Upon activation, BAX and BAK oligomerize to form large pores in the outer membrane, releasing not only cytochrome c but also full-length mtDNA into the cytosol (33). In addition, voltage-dependent anion channel 1 (VDAC1) oligomerization can cooperate with mPTP opening to mediate mtDNA release, but this mechanism has been reported mainly in non-cardiac models (3); its existence and relative contribution in cardiomyocytes await direct validation. A recent comprehensive review illustrated multiple mtDNA release routes, including herniation of the inner mitochondrial membrane, VDAC oligomerization, mPTP leakage, and mitochondria-derived vesicles (3).

Once mtDNA has reached the cytosol, the mechanisms that govern its export across the plasma membrane are less well defined but are beginning to emerge. In pyroptotic cells, gasdermin D (GSDMD) pores represent a well-established conduit for cytosolic contents, including mtDNA, to be released extracellularly. GSDMD-mediated pyroptosis has been documented after myocardial infarction, and inhibition of GSDMD palmitoylation reduces infarct size and improves cardiac function (34, 35). However, the extent of cardiomyocyte pyroptosis following myocardial infarction and its overall contribution to mtDNA release remain to be quantitatively assessed. Moreover, whether GSDMD pores directly mediate mtDNA extrusion in cardiomyocytes under non-pyroptotic conditions is still unclear.

Apart from pore-mediated direct release, mtDNA can also be exported via vesicular pathways. Mitochondria-derived vesicles (MDVs) are 70–150 nm vesicles that bud directly from mitochondria and can be secreted as extracellular vesicles (36). In adult cardiomyocytes, MDVs respond to various acute physiological and pathological stimuli, and exogenous MDVs alter stress-related gene expression, suggesting that they may act as sentinels of mitochondrial stress (36, 85). Electron microscopy has revealed that cardiomyocytes eject dysfunctional intact mitochondria and mitochondrial components within larger membranous particles, termed “cardiac exophers,” under both physiological and stressed conditions (37). These exophers are recognized and cleared by cardiac macrophages, forming a network that maintains mitochondrial homeostasis in the heart (38, 38). Notably, whether MDVs and exophers correspond to the Ambra1^+^ sEVs (Mode 2) or mt-sEVs (Mode 3) described in Section 4.2 remains to be directly demonstrated; nevertheless, vesicular export of mtDNA undoubtedly represents an important substrate for intercellular communication.

In summary, multiple redundant pathways enable mtDNA to exit donor cells – ranging from passive leakage through permeabilized membranes to active vesicular packaging. The relative contribution of each pathway during distinct phases of post-infarction remodeling remains poorly understood and represents a key knowledge gap. Once released into the extracellular space, mtDNA can exist in various forms – including naked DNA, MDVs/exophers, and intact mitochondria – thereby providing potential substrates for intercellular transfer (see Section 4.2).

## 3 Determining the activation threshold: cell-type specificity of the cGAS-STING pathway

### 3.1 Classical activation mechanism: from mtDNA recognition to signal transduction

cGAS recognizes cytosolic double-stranded DNA (including oxidatively modified mtDNA) and catalyzes the synthesis of cGAMP. cGAMP then binds STING and induces its translocation from the endoplasmic reticulum to the Golgi apparatus, which can lead to the phosphorylation of IRF3 and NF-κB via TBK1, thereby contributing to the initiation of the transcription of type I interferons and pro-inflammatory cytokines (2). In post-infarction remodeling, this pathway is hyperactivated by mtDNA, driving sterile inflammation, cell death, and fibrosis. The activation threshold is defined as the minimal mtDNA signal intensity required to activate the cGAS-STING pathway in the cytoplasm of recipient cells; its height is determined by the expression levels of cGAS/STING, co-factors, and post-translational modifications.

The efficiency of mtDNA recognition by cGAS is influenced by the degree of mtDNA oxidation. Studies suggest that oxidatively modified mtDNA may have enhanced binding affinity for cGAS (4), although this mechanism awaits direct validation. This concept echoes the “oxidative modification lowers the release threshold” described in Section 2.3—oxidized mtDNA is not only more prone to leakage from mitochondria (lowering the release threshold) but also more readily recognized by cGAS (lowering the activation threshold), thereby synergistically amplifying the pathological activation of the cGAS-STING axis.

The activation threshold of the cGAS-STING pathway differs markedly among cell types (e.g., low in macrophages, high in fibroblasts), which determines the initiation order and intensity of pathological processes such as inflammation and fibrosis, as detailed in Section 3.3.

### 3.2 Evidence chain for cGAS-STING activation after myocardial infarction

Multiple lines of evidence from different levels have supported the activation and pathological significance of the cGAS-STING pathway after myocardial infarction, forming a complete evidence hierarchy from protein expression to functional validation [Strong evidence]. Regarding the temporal dynamics, STING activation after myocardial infarction exhibits distinct phase-specific characteristics: in the acute phase (1–3 days), STING phosphorylation peaks rapidly, coinciding with massive macrophage infiltration and the peak release of inflammatory cytokines; in the subacute phase (3–7 days), the activation level gradually declines but remains relatively high; in the chronic phase (>7 days), it shows persistent low-grade activation, possibly mediated by fibroblasts and endothelial cells (4, 39). At the protein expression level, in myocardial infarction and pressure overload-induced cardiac remodeling models, the phosphorylation levels of STING and its downstream effectors TBK1 and IRF3 are markedly upregulated (11, 40). Further studies have shown that inducible nitric oxide synthase (iNOS) is upregulated in pressure-overloaded hearts, activating the cGAS-STING pathway by promoting mtDNA release, a phenomenon also validated in human hypertensive hearts (41). This further supports that mtDNA leakage (reduction of the release threshold) is an upstream event of cGAS-STING activation. Genetic knockout evidence: global STING knockout significantly attenuates pressure overload-induced cardiac remodeling, improves cardiac function, and reduces inflammation, thereby supporting the concept that STING is a key regulatory molecule in pathological remodeling (11). Cell-specific knockout evidence: to clarify the pathological contribution of STING in different cell types, macrophage-specific STING knockout has been shown to significantly reduce the inflammatory response after myocardial infarction and decrease M1 macrophage infiltration; whereas cardiomyocyte-specific STING knockout (in a diabetic cardiomyopathy model) alleviates cardiac remodeling and functional deterioration. These findings reveal the distinct pathological roles of STING in immune cells and parenchymal cells, providing a theoretical basis for cell-specific interventions (4). Gain-of-function evidence: from the perspective of enhancing mitochondrial quality control, PINK1 or Parkin overexpression suppresses cGAS-STING activation by reducing mtDNA leakage (see Section 2.1), further validating the driving role of this pathway in remodeling (1, 12).

The above evidence hierarchy—from protein expression changes to global knockout, cell-specific knockout, and gain-of-function validation—strongly supports the central role of the cGAS-STING pathway in post-infarction remodeling. As a key hub for mtDNA-mediated sterile inflammation, this pathway connects mitochondrial injury to downstream inflammatory responses, providing a solid genetic and pharmacological foundation for therapeutic strategies targeting this pathway. It is worth noting that the above evidence mainly originates from whole-tissue or macrophage/cardiomyocyte-specific models, indicating that the contribution of STING varies among cell types. Moreover, the activation threshold of STING differs significantly among cell types (see Section 3.3), which will be detailed in terms of cell-type specificity and pathological implications.

While STING serves as a central upstream integrator, the downstream effector pathways exhibit marked cell−type specificity. A recent spatial transcriptomics study reported that macrophage−specific Irf3 knockout did not attenuate cardiac inflammation on day 3 after myocardial infarction; instead, cardiomyocyte−selective deletion of Irf3 abrogated the type−I interferon response in the infarct borderzone. Importantly, this response was driven by mechanical stress−induced nuclear rupture and chromosomal DNA escape from cardiomyocytes, rather than by mtDNA (42). The study did not assess mtDNA release, cGAS−STING activation in macrophages, or the NF−κB pathway. These findings do not directly address the role of mtDNA in macrophage activation, which is the central focus of the three−threshold model. It remains possible that macrophage cGAS−STING is activated by mtDNA (either naked or vesicle−associated) after myocardial infarction, independent of the nuclear DNA−IRF3 axis described by Ninh et al. (42). However, direct experimental evidence for such activation is currently lacking. One interpretation is that IRF3−dependent type−I interferon responses are primarily mediated by cardiomyocytes in response to nuclear DNA, whereas NF−κB−driven inflammatory responses in macrophages may be triggered by mtDNA via cGAS−STING. The pathogenic role of IRF3 activation in cardiomyocytes has been further supported by independent evidence showing that cardiomyocyte−specific IRF3 deficiency ameliorates ischemic contractile dysfunction and that IRF3 activation impairs mitochondrial oxidative function, promotes inflammatory fibrotic responses, and drives heart failure progression (43). Additionally, mechanistic studies have demonstrated that STING downstream signaling exhibits cell−type−specific divergence: IRF3 activation is highly dependent on TBK1 kinase activity, whereas NF−κB responses are mediated redundantly by TBK1 and IKKϵ and are less sensitive to TBK1/IKKϵ inhibition (44). However, this proposed cell−type−specific division of labor remains hypothetical and lacks direct experimental validation. Alternative interpretations, such as redundancy between DNA sensors, cross−talk between cell types, or differential kinetics of nuclear versus mitochondrial DNA release, should also be considered. Thus, while the Ninh et al. study does not contradict the three−threshold model, it highlights the need for further investigation into the specific roles of mtDNA and nuclear DNA in post−infarction inflammation. Detailed future research directions to address these outstanding questions are provided in Section 7.2.

For a detailed summary of evidence strength, key studies, and experimental models for each core finding, see **Supplementary Table 1**.

### 3.3 Cell-type specific expression and function: determinants of the activation threshold

The expression levels, activation thresholds, and downstream effects of STING vary markedly among different cell types, which determines that the same mtDNA signal elicits distinct biological responses in different cells. The activation threshold is conceptually defined as the minimal signal intensity required to trigger cGAS-STING pathway activation after a certain concentration of mtDNA is reached in the cytoplasm of recipient cells; its height is co-determined by the expression levels of STING/cGAS, co-factors (e.g., SIRT6, IFI16), and post-translational modifications (e.g., palmitoylation, ubiquitination). This definition is qualitative; precise quantification of threshold differences among cell types awaits systematic dose-response studies. It is important to emphasize that the activation threshold is conceptually distinct from the “transmission threshold” described in Chapter 4: the transmission threshold addresses whether mtDNA “can enter the cytoplasm”, whereas the activation threshold addresses whether “entry into the cytoplasm can trigger a response”. This section systematically elaborates the differences in activation thresholds among macrophages, cardiomyocytes, endothelial cells, and fibroblasts and their pathological implications; specific pharmacological intervention strategies will be discussed in detail in Chapter 6.

Macrophages have the highest STING expression level and the lowest activation threshold, making them the primary responders to mtDNA signals. After myocardial infarction, downregulation of SIRT6 (sirtuin 6) may further lowers their activation threshold, which may enable macrophages to rapidly initiate M1 polarization even with limited mtDNA leakage (45). Genetic evidence shows that macrophage-specific *Sting1* knockout significantly reduces myocardial inflammation and ventricular remodeling (28), consistent with the findings in Section 3.2 that macrophage-specific knockout ameliorates post-infarction inflammation. Moreover, IFI16 cooperates with cGAS in macrophages to promote cGAMP production and downstream signaling (46), while STING itself can promote IFI16 degradation via ubiquitination, forming a negative feedback loop (47).

Cardiomyocytes have a moderate STING expression level and a moderate activation threshold, being activated only upon substantial mtDNA leakage or sustained pressure overload. This pathway mainly drives cardiomyocyte hypertrophy through NF-κB-mediated inflammatory responses (48). STING overexpression exerts a protective effect by inhibiting autophagy via phosphorylation of ULK1 (unc-51 like autophagy activating kinase 1) at Ser757 (49), whereas NLRX1 (NLR family member X1) alleviates endoplasmic reticulum stress and raises the activation threshold by inhibiting STING phosphorylation (50). Ubiquitin-specific protease 20 (USP20) negatively regulates STING signaling by deubiquitinating p62 and promoting autophagic degradation of STING (45). In ischemia-reperfusion injury, STING directly targets GPX4 (glutathione peroxidase 4) to induce ferroptosis (51). Cardiomyocyte-specific STING knockout (in a diabetic cardiomyopathy model) alleviates remodeling, further supporting the pathological role of cardiomyocyte STING (see Section 3.2).

Endothelial cells have STING expression levels similar to those of cardiomyocytes, but their activation pathways are more diverse. Endothelial cells can recognize exogenous mitochondrial DNA via IFI16 in a manner that is not fully blocked by cGAS inhibition, suggesting a possible cGAS−independent pathway (7)—an endothelial-specific non-canonical activation pathway distinct from the role of IFI16 as a cGAS co-factor in macrophages. In addition, heme can directly act as a STING ligand to induce endothelial senescence (52). Upon activation, STING induces CD38 (cluster of differentiation 38) expression via IRF3, leading to NAD depletion and mitochondrial dysfunction (53), or secretes IL-6 to remotely induce cardiomyocyte hypertrophy (54).

Fibroblasts have the lowest STING expression level and the highest activation threshold. Even if internalized, naked mtDNA cannot reach the cytoplasmic concentration required for activation. Therefore, fibroblast activation depends on cardiomyocyte-derived Ambra1^+^ small extracellular vesicles (sEVs). As described in Section 4.2, these vesicles deliver high-concentration mtDNA to overcome the high activation threshold of fibroblasts, thereby activating cGAS-STING and initiating fibrosis (5). Mitophagy deficiency exacerbates this process (11). The molecular mechanism underlying low STING expression in fibroblasts remains unclear and represents an important direction for future research. It must be noted that the high activation threshold of fibroblasts is inferred from indirect evidence (e.g., failure of naked mtDNA to activate them), and direct measurement of STING sensitivity in primary cardiac fibroblasts is lacking.

The above differences in activation thresholds determine the temporal sequence and functions of various cells in post-infarction remodeling: macrophages act as low-threshold “sentinels” initiating acute inflammation; cardiomyocytes and endothelial cells serve as medium-threshold executors, mediating cell death and vascular dysfunction respectively upon cumulative damage; fibroblasts act as high-threshold executors, initiating fibrotic repair only under severe injury (via high-concentration signals delivered by vesicles). Accordingly, STING signaling should be selectively targeted in different pathological phases—inhibiting macrophage STING in the acute phase to attenuate inflammation, and inhibiting fibroblast STING in the chronic phase to delay fibrosis.

This “activation threshold”, together with the “release threshold” (determining the amount of mtDNA leakage) in Chapter 2 and the “transmission threshold” (determining the efficiency of mtDNA delivery) in Chapter 4, constitutes a complete three-threshold regulatory network. Lowering of the release threshold provides more “signal raw material” for activation; lowering of the transmission threshold allows signals to efficiently reach high-threshold cells; and lowering of the activation threshold makes cells respond strongly to low-level signals. The coupling of the three thresholds determines the initiation, amplification, and irreversible progression of post-infarction remodeling. Pharmacological intervention strategies targeting these cell-type-specific pathways (e.g., STING inhibitors, curcumol, nano-decoy) will be discussed in detail in Chapter 6.

## 4 Transmission threshold: intercellular mtDNA transfer networks

### 4.1 Concept and determinants of the transmission threshold

In the preceding two chapters, we discussed the “release threshold” for mtDNA escape from mitochondria into the cytosol (Chapter 2) and the “activation threshold” for cGAS-STING pathway responses in recipient cells (Chapter 3). After being released from donor cells, mtDNA must cross the extracellular space and enter the cytoplasm of recipient cells to trigger downstream signal transduction. This process involves another critical regulatory node—the transmission threshold. The transmission threshold is conceptually defined as the sum of physical and biochemical barriers that mtDNA molecules must overcome from donor cell release to internalization by recipient cells and subsequent escape into the cytoplasm. The height of this threshold determines whether the mtDNA signal can be successfully transmitted from donor to recipient cells, serving as a bridge between intracellular release and intracellular activation. It should be emphasized that the transmission threshold is independent of the STING/cGAS expression level (i.e., the activation threshold) in recipient cells; the two concepts are strictly distinct. This concept is primarily derived from *in vitro* uptake and endosomal escape studies; its *in vivo* relevance and quantitative parameters remain to be established.

The transmission threshold is co-determined by three interrelated core factors. First, the extracellular stability of mtDNA mainly depends on its signal carrier form: naked mtDNA lacks lipid protection, is susceptible to nuclease degradation, and requires a high local concentration to be captured, resulting in a high transmission threshold; vesicle-encapsulated mtDNA resists nuclease attack and greatly lowers the transmission threshold; intact mitochondria released under severe injury have the highest transmission threshold because of their large size and requirement for specialized uptake mechanisms. Second, the uptake efficiency of recipient cells varies markedly among cell types—macrophages efficiently phagocytose naked mtDNA, fibroblasts mainly internalize vesicles via endocytosis, and endothelial cells rely on IFI16-mediated recognition to take up intact mitochondria. Damage-associated molecules such as HMGB1 (high-mobility group **Box 1**) can shift the uptake route from inefficient macropinocytosis to efficient receptor-mediated endocytosis, thereby lowering the transmission threshold (55). Third, endosomal escape capacity is the “last mile” that determines whether mtDNA can exert its signaling function. After internalization, mtDNA is usually trapped in endosomal or phagosomal compartments and must escape into the cytosol to be recognized by cytosolic sensors such as cGAS (56, 57). In myocardial infarction-related studies, mtDNA phagocytosed by macrophages successfully activates cGAS-STING (4), and mtDNA in Ambra1^+^ sEVs internalized by fibroblasts also requires escape into the cytosol to activate the pathway (5); however, the specific molecular mechanisms remain unclear. At present, the molecular machinery underlying mtDNA-specific endosomal escape is unknown, representing a core knowledge gap and an important direction for future research.

Box 1 **Limitations of the proposed three−threshold model.**

The conceptual framework presented in this review has several important limitations that should be considered when interpreting the model:

Lack of quantitative definitions: The exact numerical values or concentration ranges for the release, transmission, and activation thresholds have not been experimentally established. The three state classification (physiological homeostasis, compensation, decompensation) remains speculative.

Unknown endosomal escape machinery in the transmission threshold: While endosomal escape is postulated as a critical determinant of the transmission threshold, the specific molecular mechanisms that allow mtDNA to escape from endosomes/lysosomes into the cytosol are completely unknown.

Limited functional validation of cell type specific activation thresholds: The reported differences in activation thresholds among macrophages, cardiomyocytes, endothelial cells, and fibroblasts are largely inferred from transcriptomic data and indirect functional readouts. Direct dose response measurements of STING sensitivity in primary cardiac cells are lacking.

Extrapolation of therapeutic evidence: Several preclinical strategies discussed in Section 6 (e.g., Nrf2 activators, mito TEMPO, PCSK9 inhibitors) have been tested predominantly in non myocardial infarction models (e.g., sepsis, diabetes, liver I/R). Their efficacy and safety in post infarction remodeling remain to be directly validated.

No systematic perturbation experiments: The predicted interdependencies among the three thresholds (e.g., compensatory changes when one threshold is genetically or pharmacologically manipulated) have not been experimentally tested. Direct causal evidence linking threshold modulation to disease outcomes is still missing.

These limitations underscore that the three threshold model is a hypothesis generating synthesis intended to guide future research, rather than an established paradigm.

**Figure 1:**
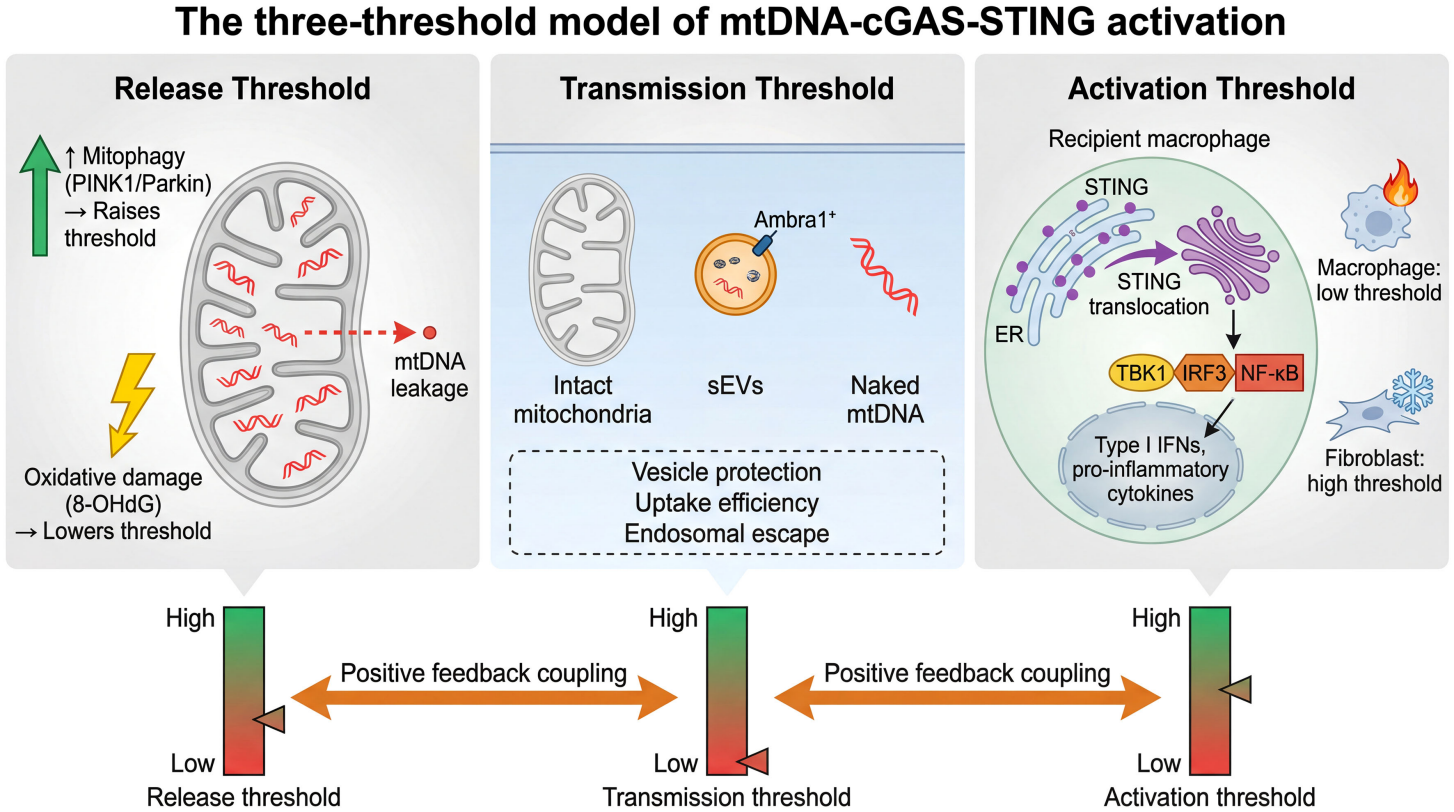
The three-threshold model of mtDNA-cGAS-STING activation. Left panel (release threshold): mtDNA leakage from mitochondria is controlled by mitophagy (PINK1/Parkin, green upward arrow) and oxidative modification (8−OHdG, yellow lightning). Middle panel (transmission threshold): Intercellular mtDNA transfer occurs via three major carriers – naked mtDNA, Ambra1^+^ sEVs, and intact mitochondria – whose efficiency depends on vesicle protection, uptake efficiency, and endosomal escape. Right panel (activation threshold): In recipient cells (macrophage shown), STING (purple) translocate from the ER to the Golgi, activating TBK1/IRF3/NF−κB. Different cell types exhibit distinct activation thresholds (macrophages: low; fibroblasts: high). Bottom: The three thresholds are interconnected by positive feedback loops, as indicated by bidirectional orange arrows. Solid arrows indicate well−established pathways; dashed arrows indicate proposed mechanisms that require experimental confirmation.

**Figure 2:**
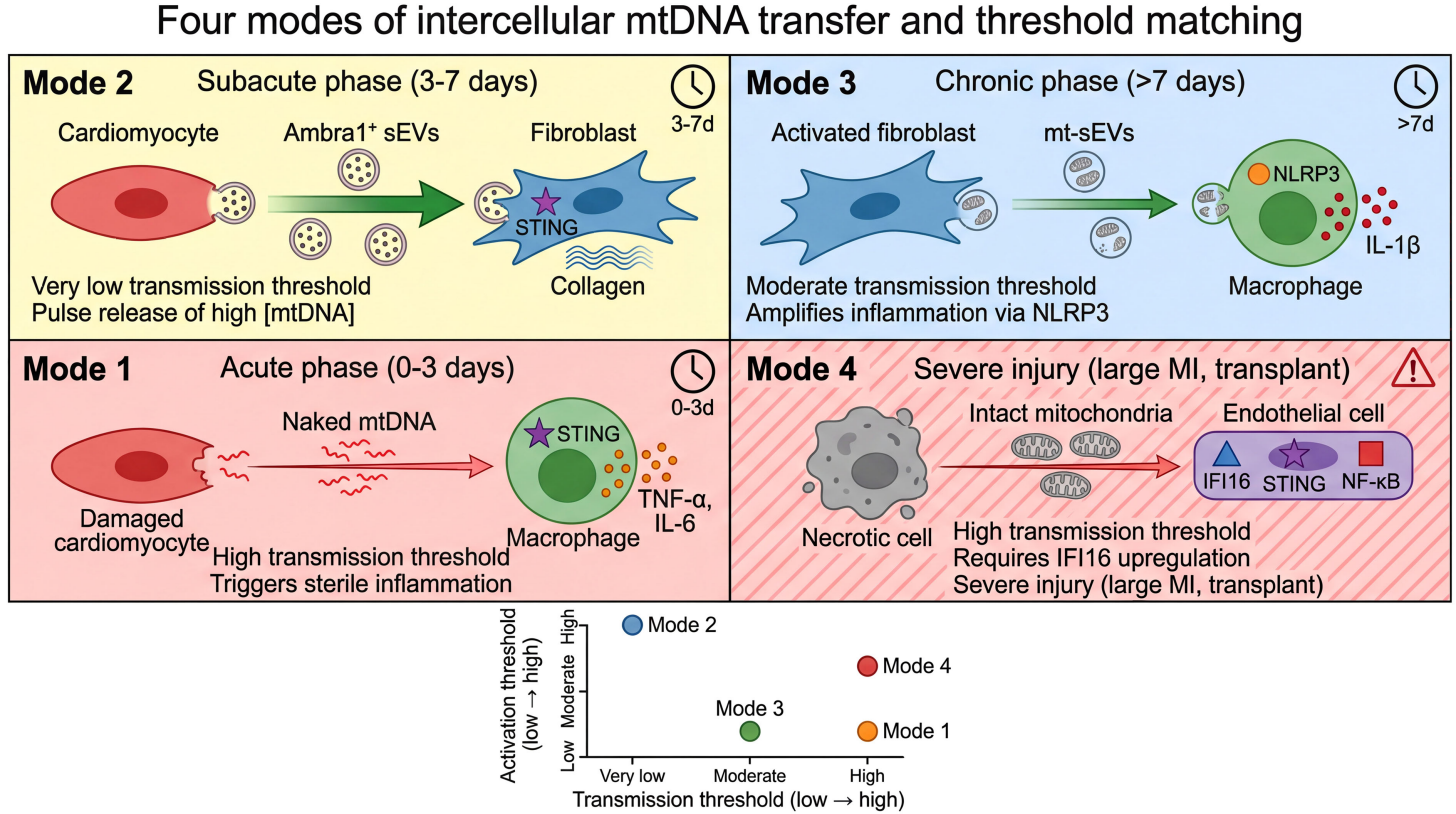
Four modes of intercellular mtDNA transfer and threshold matching. The four quadrants are arranged by increasing transmission threshold (top-left to bottom-right). Background colors indicate time windows: acute phase (light red, 0–3 days), subacute phase (light yellow, 3–7 days), chronic phase (light blue, >7 days). Mode 2: Cardiomyocytes release Ambra1^+^ sEVs delivering high-concentration mtDNA to fibroblasts, overcoming their high activation threshold via pulse release (subacute phase). Mode 3: Activated fibroblasts shed mt-sEVs that activate macrophages via NLRP3, amplifying inflammation (chronic phase). Mode 1: Naked mtDNA from damaged cardiomyocytes triggers macrophage STING activation, initiating sterile inflammation (acute phase). Mode 4: Intact mitochondria from necrotic cells activate endothelial cells via IFI16-STING, promoting vascular inflammation (severe injury). Bottom scatter plot: Each mode’s transmission threshold plotted against the recipient cell’s activation threshold; the dashed diagonal line illustrates threshold matching. The indicated modes represent the predominant transfer pathways during each time window; however, concurrent or overlapping transfer modes may also occur. The threshold matching scatter plot is based on qualitative data; quantitative validation is needed.

**Figure 3:**
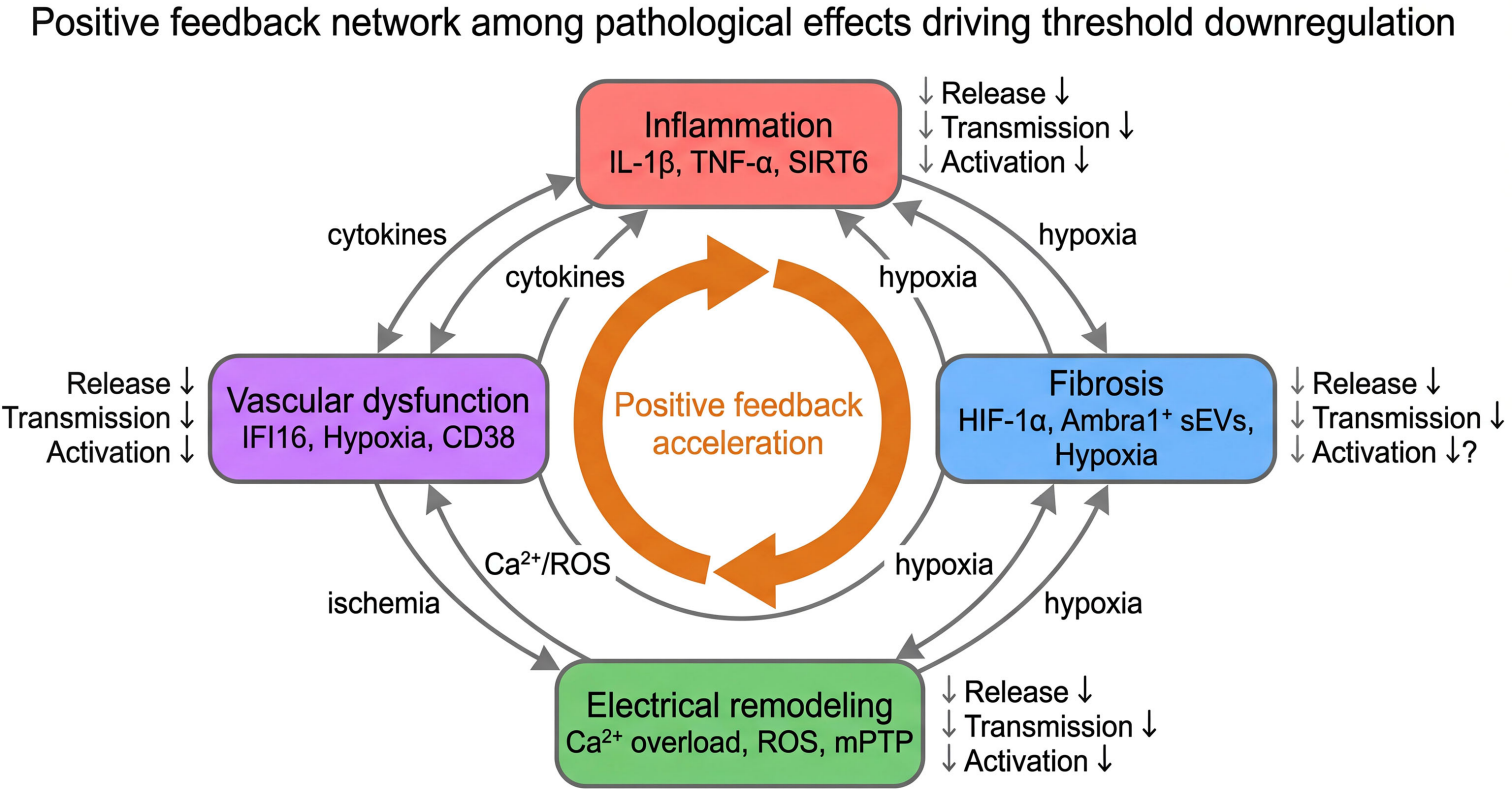
Positive feedback network among pathological effects driving threshold downregulation. Four pathological effects (inflammation, fibrosis, electrical remodeling, and vascular dysfunction) are interconnected by arrows indicating mutual amplification. Each effect reduces the release, transmission, and (except for fibrosis) activation thresholds, as indicated by downward arrows. The central circular arrow denotes the self-accelerating nature of the network. Once the three thresholds fall below a critical point, post-infarction remodeling becomes irreversible. Detailed mechanisms are described in Sections 5.1–5.4. The interconnections are based on indirect evidence; direct experimental proof of each cross−talk direction is lacking.

**Figure 1** summarizes the three−threshold model, which integrates the release, transmission, and activation thresholds into a unified framework. It should be noted that **Figures 1**–**3** are conceptual illustrations intended to summarize the proposed framework; they do not represent quantitative models. Based on the above factors, intercellular mtDNA transfer can be summarized into four main modes, which differ in signal carrier, transmission threshold, and key regulatory molecules, as shown in **Table 1**. Vesicle-encapsulated mtDNA has the lowest transmission threshold due to protection and targeted delivery. Notably, as shown in **Table 2** of Section 3.3, fibroblasts have an extremely high activation threshold, and even if internalized, naked mtDNA cannot reach the cytoplasmic concentration required for activation. The reason why vesicle-mediated transfer can effectively activate fibroblasts is not only that vesicles lower the transmission threshold (protection + efficient endocytosis) but, more critically, that fusion of vesicles with endo/lysosomal membranes releases the encapsulated high-concentration mtDNA in a “pulse” into the cytoplasm, instantaneously raising the local mtDNA concentration above the high activation threshold of fibroblasts (see Section 4.2). This mechanism reveals a synergistic regulatory relationship between different thresholds—lowering the transmission threshold enables high-activation-threshold cells to respond, but final activation still depends on the signal strength provided by the vesicles. Identifying specific phagocytic receptors for mtDNA and elucidating the mechanisms of endosomal escape are important future research directions.

**Table 1:** Four modes of intercellular mtDNA transfer.

Mode	Donor → recipient	Signal carrier	Transmission threshold	Main determinants	Key regulatory molecules	Evidence strength
1	Cardiomyocyte → Macrophage	Naked mtDNA	High	Low extracellular stability; uptake depends on HMGB1	HMGB1/TLR2/4	Moderate (single study, diabetic atrial fibrillation model)
2	Cardiomyocyte → Fibroblast	Ambra1^+^ sEVs	Very low	Vesicle protection, efficient endocytosis, membrane fusion-mediated pulse release*	Ambra1	Moderate (single study; awaits independent validation)
3	Fibroblast → Macrophage	mt-sEVs	Moderate	Vesicle protection; Bnip3-regulated release	Bnip3†	Weak/Preliminary (single study + conference abstract)
4	Injured cell → Endothelial cell	Intact mitochondria	High	Large particle size; requires high IFI16 expression	IFI16	Moderate (transplant rejection model; direct cardiac evidence limited)

*The membrane fusion-mediated high-concentration mtDNA pulse release in Mode 2 is key to overcoming the high activation threshold of fibroblasts; this mechanism is mainly based on a single study (5) and awaits independent validation. †The regulatory role of Bnip3 (BCL2/adenovirus E1B 19 kDa protein-interacting protein 3) in Mode 3 is based on a conference abstract (58) and awaits formal study confirmation. The role of IFI16 in Mode 4 mainly comes from a transplant rejection model (7); direct evidence in the heart remains to be accumulated. Evidence strength definitions: see **Table 2** note.

**Table 2:** Response characteristics of STING in different cell types.

Cell type	STING expression level	Activation threshold	Major effects upon activation	Specific regulatory factors	Evidence strength
Macrophage	High	Low	M1 polarization, pro-inflammatory cytokine storm	SIRT6 (stabilizes STING), IFI16 (synergizes with cGAS)	Strong
Cardiomyocyte	Moderate	Moderate	Apoptosis, ferroptosis, autophagy regulation	NLRX1 (inhibits STING phosphorylation), USP20 (promotes STING degradation)	Moderate
Endothelial cell	Moderate	Moderate (diverse activation pathways)	Upregulation of adhesion molecules, IL-6 secretion, NAD depletion	IFI16 (independent mtDNA recognition), CD38 (mediates NAD depletion)	Moderate
Fibroblast	Low	High	Activation, collagen deposition	To be identified (activation depends on transmission mechanism, see Chapter 4)	Moderate (inferred from indirect evidence)

The specific regulatory factors for fibroblasts have not yet been identified; their activation depends on cardiomyocyte−derived Ambra1^+^ sEVs (see Chapter 4). Endothelial cells exhibit diverse activation pathways, including the IFI16−dependent non−canonical pathway. Evidence strength definitions (consistent for **Tables 2**, **1**, **4**): Strong: validated by multiple independent teams in MI/I/R models. Moderate: single−team report, or derived from non−MI models but mechanistically relevant, or inferred from indirect evidence. Weak/Preliminary: single study or conference abstract, key steps awaiting confirmation. Hypothesis: no experimental validation (used only in **Table 4** for combination strategies).

### 4.2 Four modes of intercellular mtDNA transfer

Based on the transmission threshold framework established in Section 4.1, mtDNA is mainly transferred between cells through four modes after myocardial infarction. In order of increasing transmission threshold, they are: Mode 2 (cardiomyocyte → fibroblast, very low threshold), Mode 3 (fibroblast → macrophage, moderate threshold), Mode 1 (cardiomyocyte → macrophage, high threshold), and Mode 4 (injured cell → endothelial cell, high threshold with requirement for IFI16 upregulation). Each is described below.

Mode 2 (very low transmission threshold): cardiomyocyte → fibroblast, mediated by Ambra1^+^ sEVs. Cardiomyocytes release Ambra1^+^ small extracellular vesicles (sEVs) carrying mtDNA via secretory autophagy, which efficiently deliver mtDNA to fibroblasts. These vesicles not only protect mtDNA from extracellular nuclease degradation but also release the encapsulated high-concentration mtDNA in a “pulse” into the cytoplasm through membrane fusion, instantaneously exceeding the extremely high activation threshold of fibroblasts (Section 3.3). This activates the cGAS-STING pathway, drives fibroblast-to-myofibroblast conversion, and promotes fibrosis (5). Cardiac-specific downregulation of Ambra1 inhibits this process and alleviates ischemic cardiac fibrosis. Currently, this mechanism is mainly based on a single study and awaits independent validation [Moderate evidence]. This mode is a key step in the initiation of post-infarction fibrosis and dominates the subacute phase (3–7 days).

Mode 3 (moderate transmission threshold): fibroblast → macrophage, mediated by mt-sEVs. Activated fibroblasts release mitochondria-enriched small extracellular vesicles (mt-sEVs), which are taken up by macrophages, leading to increased expression of IL-1β and IL-6 and amplification of the inflammatory response (6). Myocardial injection of fibroblast-derived mt-sEVs worsens cardiac dysfunction, an effect reversed by the NLRP3 inhibitor CY-09 (6). Currently, this mode is mainly based on a single study. Regarding upstream mechanisms, the mitophagy receptor Bnip3 (BCL2/adenovirus E1B 19 kDa protein-interacting protein 3) is upregulated in infarct-zone fibroblasts and may mediate mt-sEV release (58)(conference abstract), but the direct link between Bnip3 and the cGAS-STING pathway remains to be confirmed [Weak/Preliminary evidence].

It remains unresolved whether the mt-sEVs in Mode 3 directly activate the cGAS-STING pathway, act primarily through the NLRP3 inflammasome, or engage both in a cooperative manner. Notably, the current evidence for Mode 3 is largely based on the finding that fibroblast-derived mt-sEVs promote inflammation and ventricular remodeling via the NLRP3 pathway in macrophages, as demonstrated by Zhao et al. (6) using the specific NLRP3 inhibitor CY-09. However, cGAS-STING signaling and NLRP3 inflammasome activation are not mutually exclusive. Recent reviews have highlighted that cGAS-STING and NLRP3 are functionally interconnected; for instance, cGAS-STING activation can potentiate NLRP3 inflammasome-mediated pyroptosis, and both pathways contribute to cardiovascular inflammation (59, 60). Synergy between the two pathways has also been observed in an intestinal ischemia-reperfusion model (31), although direct evidence in the heart is still lacking.

Thus, Mode 3 may involve a parallel or converging network that integrates both pathways, which is not fully captured by the current three-threshold model centered on the cGAS-STING axis. Caution should be exercised when incorporating this mode into the model, and future studies using cGAS or STING knockout cells or animal models are required to definitively determine the dependency of Mode 3 on the cGAS-STING pathway. This mode sustains inflammation during the chronic phase (>7 days), driving the progression of remodeling.

Mode 1 (high transmission threshold): cardiomyocyte → macrophage, mediated by naked mtDNA.

Damaged cardiomyocytes release free mtDNA into the extracellular space, which is phagocytosed by macrophages and activates the cGAS-STING pathway, promoting M1 polarization and pro-inflammatory cytokine release (4). Naked mtDNA lacks protection, is susceptible to nuclease degradation, and requires a high local concentration to be captured, resulting in a high transmission threshold. Macrophage uptake efficiency is regulated by HMGB1: HMGB1 shifts the uptake route from inefficient macropinocytosis to efficient receptor-mediated endocytosis via TLR2/4 (Toll-like receptors 2 and 4), thereby lowering the transmission threshold (55). In the myocardial infarction microenvironment, both necrotic cells and activated macrophages release HMGB1, which may amplify mtDNA signaling. This mode serves as the trigger for sterile inflammation after myocardial infarction and dominates the acute phase (0–3 days). Currently, this mechanism is mainly based on a study by Meng’s team in a diabetic atrial fibrillation model (4); its generalizability to pure myocardial infarction models awaits independent validation [Moderate evidence]. It should be noted that macrophages are not limited to uptake of naked mtDNA; they can also internalize vesicle−associated mtDNA, such as fibroblast−derived mt−sEVs via Mode 3 (6), and may employ other endocytic pathways to take up vesicular mtDNA. Thus, macrophages act as multimodal receivers of mtDNA signals.

Mode 4 (high transmission threshold, requiring IFI16 upregulation): injured cell → endothelial cell, mediated by intact mitochondria.

Under severe injury, intact functional mitochondria are released into the extracellular space, internalized by endothelial cells, and activate the STING-NF-κB pathway via the IFI16-dependent non-canonical pathway, promoting T cell adhesion (7). This effect is not fully blocked by cGAS inhibition and has been attributed to IFI16; however, whether IFI16 acts completely independently of cGAS or cooperates with cGAS under certain conditions remains to be definitively established through direct genetic experiments (e.g., IFI16 knockout) (7). Internalized mitochondria undergo fusion and STING-dependent mitophagy, leading to their clearance. Ex vivo heart perfusion confirmed that exogenous mitochondria can activate mouse cardiac endothelial cells. In endothelial cells, IFI16 has been reported to recognize mitochondrial DNA in a manner that is not fully blocked by cGAS inhibition, suggesting the existence of a cGAS-independent pathway (7). However, whether IFI16 is entirely independent of cGAS or works in concert with cGAS requires further investigation using IFI16 knockout models. This putative function is distinct from its role as a cGAS co-factor in macrophages (Section 3.3). This mode has a high transmission threshold for two reasons: (i) intact mitochondria are large (0.5–1 μm) and require specialized phagocytic mechanisms for internalization; (ii) under physiological conditions, IFI16 expression in endothelial cells is low, and it can be upregulated by inflammatory signals (e.g., IL-1β via m^6^A modification) or GNAQ deficiency to enable effective response (61, 62). Therefore, this mode is activated only under severe inflammation or ischemia (e.g., large-area myocardial infarction, heart transplantation), exacerbating vascular inflammation and endothelial dysfunction.

It should be clarified that the mitochondria used in therapeutic mitochondrial transplantation are carefully isolated from autologous non-ischemic tissue (e.g., skeletal muscle) under gentle conditions, and are rigorously quality-controlled to maintain intact mitochondrial membrane potential and oxidative phosphorylation capacity. These healthy exogenous mitochondria exert cardioprotective effects through mechanisms such as fusion with recipient mitochondria, replenishment of mitochondrial DNA, and restoration of ATP synthesis (63, 64). The safety and efficacy of this approach have been further validated in a prospective, triple-blinded, parallel-group, blocked randomized clinical trial (RCT) in 30 patients with acute ST-elevation myocardial infarction (STEMI), where intracoronary delivery of autologous platelet-derived mitochondria significantly improved left ventricular ejection fraction (LVEF) and exercise capacity at 40 days compared to standard of care, with no major adverse cardiac events reported (65). In contrast, mitochondria released from necrotic cells after myocardial infarction typically suffer from loss of membrane potential, cardiolipin peroxidation, and impaired respiratory chain complex activity following ischemia/reperfusion injury (24)(Buja, 2023). These damaged mitochondria act as DAMPs, activating innate immunity through pathways such as IFI16-STING and promoting inflammation. Therefore, the healthy exogenous mitochondria used in transplantation are fundamentally distinct from the pathologically released endogenous damaged mitochondria discussed in this review, and there is no contradiction.

Currently, the role of IFI16 in this context is primarily derived from a transplant rejection model (7); ex vivo heart perfusion provides supportive evidence, but *in vivo* validation in myocardial infarction models is still needed [Moderate evidence].

The four modes achieve a graded response to injury severity through matching of transmission thresholds with recipient cell activation thresholds: Mode 2 (very low transmission threshold → high activation threshold) ensures efficient fibroblast activation under severe injury; Mode 3 (moderate transmission threshold → low activation threshold) sustains inflammation; Mode 1 (high transmission threshold → low activation threshold) rapidly initiates inflammation under mild-to-moderate injury; Mode 4 (high transmission threshold plus additional conditions → moderate activation threshold) participates in vascular responses only under pathological conditions with IFI16 upregulation. This “threshold matching” mechanism ensures precise alignment of injury intensity with cellular responses, preventing excessive inflammation or inadequate repair. Switching between modes is dynamically regulated by microenvironmental signals (e.g., hypoxia, inflammatory cytokines) and cellular states (see Section 4.3). The time-window assignment reflects the predominant period for each mode; however, overlapping or concurrent activation of multiple modes may occur in the pathological setting. **Figure 2** illustrates the four modes of intercellular mtDNA transfer, arranged by increasing transmission threshold and aligned with their respective time windows.

The four modes described above represent the major transfer pathways reported in the current literature, but they may not be exhaustive. For instance, whether naked mtDNA can also be taken up by fibroblasts or endothelial cells remains to be investigated. Furthermore, gap junctions formed by connexin 43 (Cx43) have been shown to mediate intercellular transfer of small molecules and microRNAs. A growing body of evidence indicates that Cx43 can also facilitate the transfer of entire organelles, including mitochondria, through a process termed gap junction internalization, which generates double-membrane vesicles (connexosomes) that enclose these organelles (66). Whether such a mechanism could allow the transfer of intact mitochondria (which, upon degradation in recipient cells, might release mtDNA) or directly transfer mtDNA fragments between cardiomyocytes and macrophages remains an intriguing but unexplored question. In support of the multifaceted roles of Cx43 in intercellular communication, a recent review highlighted that Cx43 localized in mitochondria is involved in mitochondrial respiration, whereas Cx43 present in extracellular vesicles and tunneling nanotubes participates in long-distance information exchange (67). Moreover, another review discussed the role of the short Cx43 isoform GJA1-20k in regulating mitochondrial dynamics and homeostasis (68), suggesting an intrinsic link between Cx43 biology and mitochondrial quality control that could influence organelle transfer. Together, these observations underscore the need for future studies to directly examine whether Cx43-based intercellular channels contribute to mtDNA-mediated crosstalk in the post-infarction heart.

Context from recent studies on DNA sources. A recent spatial transcriptomics study identified cardiomyocyte nuclear DNA escape as a trigger for type−I interferon responses (42). However, that study did not assess mtDNA or cGAS−STING activation in macrophages. Thus, while the four modes described above focus on mtDNA as an intercellular messenger, the relative contribution of mtDNA versus nuclear DNA to post−infarction inflammation remains an open question. The pathways proposed in Modes 1 and 3 (macrophage uptake of naked or vesicle−associated mtDNA) may operate alongside nuclear DNA−driven responses, and future studies using myeloid−specific cGAS or STING knockouts are needed to dissect their respective roles.

### 4.3 Integration of the transmission threshold with the three-threshold model

Based on the threshold matching characteristics of the four modes described in Section 4.2, this section further integrates the transmission threshold with the release threshold (Chapter 2) and the activation threshold (Chapter 3). Based on the evidence summarized in previous sections, the three thresholds are hypothesized to not operate independently but rather to be interdependent and progressively linked: the release threshold determines the amount of mtDNA leakage; the transmission threshold determines whether the leaked mtDNA can cross the extracellular space and enter the recipient cell cytoplasm; and the activation threshold determines whether mtDNA that has entered the cytoplasm can trigger downstream signal transduction. Among the four modes, Mode 2 (very low transmission threshold → high activation threshold) and Mode 1 (high transmission threshold → low activation threshold) exemplify the complementary matching between transmission and activation thresholds, which is the core mechanism for graded responses to injury severity. (see **Figure 2**; **Table 1**) This interdependent relationship, while conceptually appealing, awaits experimental confirmation (e.g., by manipulating one threshold and measuring compensatory changes in the others).

Lowering of the release threshold provides more “raw material” for transmission; lowering of the transmission threshold enables signals to efficiently reach high-threshold cells; and lowering of the activation threshold makes cells respond strongly to low-level signals. An abnormality in any one threshold can be partially compensated by the others, but compensation is limited and ultimately still drives pathological remodeling. For example, in a myocardial ischemia-reperfusion injury model, Parkin-mediated mitophagy defects lower the release threshold (12), while Ambra1^+^ sEVs released by damaged cardiomyocytes lower the transmission threshold (5), together promoting fibroblast and macrophage activation. Therefore, targeting any single node among the three thresholds can inhibit overactivation of this axis, and simultaneously targeting multiple thresholds (e.g., mito-TEMPO to reduce mtDNA oxidation combined with H-151 to inhibit STING) may produce synergistic effects (see Section 6.4).

## 5 Downstream effects: from cellular crosstalk to tissue remodeling

### 5.1 Inflammatory amplification and immune microenvironment remodeling

This section focuses on macrophage STING activation mediated by Mode 1 (cardiomyocyte → macrophage naked mtDNA transfer) and Mode 3 (fibroblast → macrophage mt-sEV transfer), and elaborates the positive feedback loop through which the release and activation thresholds are mutually downregulated.

After myocardial infarction, mtDNA released from necrotic cardiomyocytes is rapidly recognized by macrophages infiltrating the infarct zone, activating the cGAS-STING pathway and initiating sterile inflammation. M1 macrophages release large amounts of pro-inflammatory cytokines (TNF-α, IL-1β, IL-6), which directly damage cardiomyocytes and recruit more immune cells, forming a positive feedback amplification loop (69, 70).

As described in Sections 3.2 and 3.3, macrophage STING signaling plays an important role in driving M1 polarization, and SIRT6 further lowers the activation threshold by stabilizing the STING protein (39, 71). Moreover, in Mode 3, activated fibroblasts release mt-sEVs that are also taken up by macrophages and activate cGAS-STING, establishing a fibroblast-macrophage positive feedback loop that further amplifies inflammation (see Section 4.2).

Pro-inflammatory cytokines released by M1 macrophages (especially IL-1β and TNF-α) directly damage cardiomyocytes. In a myocardial ischemia-reperfusion injury model, activation of the caspase-1/IL-1β axis has been shown to cause mitochondrial homeostasis imbalance and cardiomyocyte injury (72). In turn, mtDNA released from damaged cardiomyocytes can be taken up by cardiomyocytes themselves and, through binding to nucleolin, upregulate the expression of IL-1β and TNF-α (73), forming a positive feedback amplification loop. This process lowers the release threshold (Chapter 2); simultaneously, SIRT6-mediated stabilization of STING lowers the activation threshold (Section 3.3); and inflammation-induced HMGB1 release enhances the efficiency of mtDNA uptake by macrophages, lowering the transmission threshold (Section 4.1).

The above positive feedback loop mutually reinforces inflammation, mitochondrial damage, and threshold downregulation: inflammatory cytokines damage cardiomyocytes → more mtDNA release → more macrophage STING activation → more inflammatory cytokines, creating a self-accelerating vicious cycle. **Table 3** systematically summarizes the effects of inflammation, fibrosis, electrical remodeling, and vascular dysfunction on the three thresholds (see Section 5.5; **Table 3**), collectively driving the early inflammatory amplification in post-infarction remodeling.

**Table 3:** Effects of pathological processes on the three thresholds.

Pathological process	Effect on release threshold	Effect on transmission threshold	Effect on activation threshold
Inflammatory amplification	Lowered	Lowered	Lowered
Fibrosis	Lowered	Lowered	Possibly lowered
Electrical remodeling	Lowered	Lowered	Lowered
Vascular dysfunction	Lowered	Lowered	Lowered

Direct evidence for a direct effect of fibrosis on the activation threshold is lacking; “possibly lowered” is based on the indirect influence of the hypoxic microenvironment on STING signaling. The basis for each effect is described in Sections 5.1–5.4.

### 5.2 Fibrosis and extracellular matrix deposition

This section focuses on fibroblast STING activation mediated by Mode 2 (cardiomyocyte → fibroblast Ambra1^+^ sEV transfer). As described in Section 4.2, cardiomyocyte-derived Ambra1^+^ sEVs drive fibrosis by activating the cGAS-STING pathway in fibroblasts (5). On this basis, the section elaborates how the fibrotic microenvironment further lowers the transmission and release thresholds through hypoxia and mechanical stress, forming a self-reinforcing positive feedback loop.

Following the inflammatory response, the fibrotic program is initiated. Fibroblasts receive dual signals from cardiomyocytes (via Mode 2) and immune cells (via TGF-β1) (5, 74). TGF-β1 (transforming growth factor-β1) is a central inducer of fibroblast activation, promoting α-SMA (alpha-smooth muscle actin) and collagen expression through the Smad2/3 pathway; its sources include M1 macrophages and autocrine secretion by fibroblasts. Whether TGF-β1 signaling directly cross-talks with the cGAS-STING pathway remains unclear, but the two act synergistically in fibroblast activation. Activated fibroblasts synthesize large amounts of type I and type III collagen, and the type I/III collagen ratio is an important determinant of myocardial stiffness. In a rat model of myocardial infarction, an increase in the type III/I collagen ratio was directly associated with improved ventricular compliance (75); moreover, *in vitro* experiments have shown that increasing the proportion of type III collagen significantly reduces matrix stiffness (76), confirming from both positive and negative sides the decisive role of the collagen ratio in myocardial mechanical properties.

The fibrotic process itself may create a positive feedback loop that further lowers the transmission and release thresholds (77). As collagen deposition increases, local hypoxia worsens, leading to stabilization of HIF-1α (hypoxia-inducible factor-1α) in cardiomyocytes and promotion of secretory autophagy, potentially resulting in more Ambra1^+^ sEV release and thus lowering of the transmission threshold (78) (see also Chapter 4). Notably, hypoxia-induced HIF-1α plays a protective “brake” role in fibroblasts by limiting mitochondrial reactive oxygen species accumulation and inhibiting excessive activation (79). In addition, collagen deposition increases mechanical stress on cardiomyocytes, which may induce mitochondrial damage and mtDNA release (awaiting direct validation), thereby lowering the release threshold (Chapter 2). TGF-β1 secreted by fibroblasts can also act back on cardiomyocytes, promoting their injury and vesicle release (74, 80).

In summary, post-infarction fibrosis is initiated by Mode 2, regulated by TGF-β/Smad signaling, and further lowers the transmission and release thresholds through hypoxia and mechanical stress, potentially forming a self-accelerating vicious cycle. It should be noted that direct evidence for a direct effect of fibrosis on the activation threshold is lacking, hence it is listed as “possibly lowered” in **Table 3** of Section 5.5 (see the table note).

### 5.3 Electrical remodeling and arrhythmias

This section focuses on how inflammatory cytokines (derived from Modes 1 and 3) and mitochondrial damage (reduced release threshold) regulate ion channels and gap junctions via the STING-NF-κB pathway, and analyzes the bidirectional regulation between electrical remodeling and the three thresholds. Electrical remodeling after myocardial infarction is characterized by alterations in ion channels and gap junction proteins, leading to slowed conduction, increased dispersion of repolarization, and providing a substrate for reentrant arrhythmias (81, 82).

Ion channel and gap junction remodeling. The sodium channel Nav1.5 is downregulated after myocardial infarction, resulting in slowed conduction velocity (83). The deubiquitinating enzyme USP38 (ubiquitin-specific protease 38) inhibits Nav1.5 expression by activating the TAK1 (transforming growth factor-β-activated kinase 1)/NF-κB pathway, and NF-κB downstream of STING can also directly suppress Nav1.5 transcription (84). In addition, hypoxia can upregulate miR-448 (microRNA-448) via the HIF-1α/NF-κB axis, thereby inhibiting SCN5A expression and sodium current, increasing arrhythmic risk (85). Downregulation of the transient outward potassium channel Kv4.3 prolongs the action potential duration (86, 87). Hyperphosphorylation of the calcium-handling protein RyR2 (ryanodine receptor 2) and downregulation of SERCA2a (sarco/endoplasmic reticulum Ca²^+^-ATPase 2a) lead to calcium overload (88). Calcium overload can activate opening of the mPTP (mitochondrial permeability transition pore), promoting mtDNA oxidation and release (89). The gap junction protein Cx43 (connexin 43) is downregulated and its distribution becomes disorganized, resulting in weakened electrical coupling (90). Recent studies have confirmed that reactive oxygen species (ROS) generation can reduce Cx43 levels by stimulating NOX2 (NADPH oxidase 2), causing gap junction dysfunction and ventricular arrhythmias (91).

Positive feedback loop between electrical remodeling and the three thresholds. Electrical remodeling and the mtDNA−cGAS−STING axis exhibit bidirectional regulation. First, calcium overload induces loss of mitochondrial membrane potential and ROS production (92), triggering opening of the mPTP (93), which in turn leads to mtDNA release into the cytosol (27), thereby lowering the release threshold (Chapter 2) [Strong evidence]. Second, as described in Section 5.1, inflammatory cytokines (TNF−α, IL−1β) persistently activate STING signaling through the USP38/TAK1/NF−κB pathway (84), thereby lowering the activation threshold (Chapter 3). Third, ventricular tachycardia/fibrillation is associated with haemodynamic collapse, which directly reduces myocardial perfusion and exacerbates ischemia (94). Ischemia, in turn, is a potent stimulus for the release of small extracellular vesicles (sEVs) from cardiomyocytes (95), as discussed in Section 4.2 (Modes 2 and 3). Consequently, this pathway may lower the transmission threshold (Chapter 4). Conversely, a lowered release threshold leads to more mtDNA leakage, further activating STING and exacerbating inflammation and ion channel inhibition; a lowered activation threshold enables even weak STING signals to trigger strong responses, forming a closed loop. These positive feedback loops likely contribute to mutually reinforce electrical remodeling, inflammation, and mitochondrial damage, potentially driving the chronic progression of arrhythmias. The relevant mechanisms are summarized in **Table 3** of Section 5.5.

### 5.4 Vascular dysfunction

This section focuses on the IFI16-STING axis activated by Mode 4 (injured cell → endothelial cell intact mitochondria transfer) and elaborates the molecular mechanisms of vascular dysfunction and its impact on the three thresholds.

As described in Section 4.2, intact mitochondria released during severe myocardial injury are internalized by endothelial cells, where IFI16 recognizes exogenous mitochondrial DNA independently of cGAS, activating the STING-NF-κB pathway, upregulating adhesion molecules, and promoting immune cell adhesion (7). STING also induces CD38 expression via IRF3, leading to NAD(P)H depletion and eNOS (endothelial nitric oxide synthase) uncoupling, thereby impairing vasodilation (53). Moreover, STING activation in endothelial cells can secrete IL-6, which remotely induces cardiomyocyte hypertrophy (54). The above endothelial dysfunction may contribute microvascular rarefaction and persistent hypoxia.

Hypoxia is widely recognized as a critical link connecting vascular dysfunction and myocardial injury (96). On one hand, hypoxia can induce mitochondrial ROS production and mPTP opening (32, 97), promoting mtDNA oxidation and release (98)(see also Section 2.3), thereby lowering the release threshold; on the other hand, hypoxia upregulates secretory autophagy in cardiomyocytes via HIF−1α, increasing the production of Ambra1^+^ sEVs (5, 99)(see also Section 4.2), thereby lowering the transmission threshold. Clinical studies have shown that microvascular dysfunction is an independent predictor of adverse remodeling after myocardial infarction (100), but the molecular mechanisms by which it directly causes mtDNA release remain to be further elucidated. The relevant mechanisms are summarized in **Table 3** of Section 5.5.

### 5.5 Positive feedback integration and sustained downregulation of the three thresholds

The pathological effects described above (inflammation, fibrosis, electrical remodeling, and vascular dysfunction) do not operate independently but continuously lower the release, transmission, and activation thresholds through three interconnected positive feedback loops (summarized in **Table 3** and illustrated in **Figure 3**).

Inflammatory loop: pro−inflammatory cytokines damage mitochondria (lowering the release threshold) and upregulate SIRT6 to stabilize STING (lowering the activation threshold) (see Section 5.1). Fibrotic loop: hypoxia promotes vesicle release (lowering the transmission threshold) and induces mitochondrial damage (lowering the release threshold) (see Section 5.2). Vascular loop: microvascular rarefaction and hypoxia lower the release and transmission thresholds, and endothelial IFI16−STING lowers the activation threshold (see Section 5.4).

Activation of any single loop may rapidly spread to the others through cytokine signaling, hypoxia, and ischemia, potentially creating a self−accelerating network effect. Once the three thresholds are persistently downregulated beyond a critical point, may remodeling enters an irreversible phase. The effects of each pathological process on the three thresholds are summarized in **Table 3**. The interconnections among these positive feedback loops are illustrated in **Figure 3**. (Figure legend already notes that interconnections are based on indirect evidence.)

## 6 Targeting the three thresholds: therapeutic strategies for post-infarction remodeling

### 6.1 Intervention concepts and translational prospects

The “three−threshold model” proposed in this review divides the regulation of the mtDNA−cGAS−STING axis into three sequential and interconnected nodes: the release threshold, the transmission threshold, and the activation threshold. Based on this model, intervention strategies can be classified as: raising the release threshold (blocking mtDNA leakage at the source), raising the transmission threshold (blocking intercellular mtDNA communication), and raising the activation threshold (inhibiting STING signaling in recipient cells).

Traditional strategies often focus on a single molecule; however, the positive feedback loops described in Chapter 5 may cause mutual downregulation of the three thresholds, potentially limiting the effectiveness of single−target interventions to completely halt the pathological process. Therefore, this review proposes a multi−node, stage−specific intervention concept: according to the dominant type of threshold breach at different phases after myocardial infarction—acute phase (0–3 days) mainly characterized by release threshold breach (massive mtDNA leakage), subacute phase (3–7 days) dominated by the transmission threshold (vesicle−mediated fibrotic signals), and chronic phase (>7 days) characterized by persistently lowered activation threshold (high STING sensitivity)—targeting the corresponding node selectively, and combining drugs to more effectively interrupt the vicious cycle.

Currently, strategies targeting the three thresholds include approved drugs, preclinical small molecules, and gene therapies, which will be detailed in Sections 6.2–6.4. Based on the time−window characteristics of post−infarction remodeling, Section 6.4 will propose stage−specific combination regimens. The three−threshold model provides a systematic framework for precision intervention in post−infarction remodeling.

Clinical translation challenges. Despite the promise of the model, several challenges remain. First, many therapeutic strategies, especially those targeting the transmission threshold, are still at the preclinical stage and lack direct validation in post−infarction models. Second, long−term complete inhibition of STING may compromise antiviral immune surveillance; therefore, intermittent use or cell−type−specific inhibition is preferable. Third, effective cell−type−specific delivery systems (e.g., AAV vectors, nanoparticles) require further optimization to avoid off−target effects and immunogenicity. Fourth, precise time−window identification depends on robust biomarkers (e.g., circulating mtDNA, MitoEVs, SIRT6), which are not yet clinically validated. Fifth, the long−term efficacy and safety of repurposed drugs such as levosimendan in chronic post−infarction remodeling remain to be evaluated. These challenges are highlighted where appropriate in the following sections, and detailed evidence strengths are provided in **Table 4**.

**Table 4:** Stage-specific intervention strategies based on time windows.

Time window	Dominant threshold	Representative strategies	Evidence strength	Remarks
Acute phase (0–3 days)	Release threshold	Levosimendan, mito-TEMPO, Nrf2 activators	Moderate (levosimendan: direct MI model; others: non-MI models)	Approved or preclinical; requires validation in MI models
Subacute phase (3–7 days)	Transmission threshold	Targeting Ambra1 (proof of concept)	Weak/Preliminary (single study, no independent validation)	No clinically available strategy yet
Chronic phase (>7 days)	Activation threshold	H-151, curcumol, PTS	Moderate (H-151: multi-team; natural products: single studies)	Need to address bioavailability/safety
All phases	Combination targeting	mito-TEMPO + H-151	Hypothesis (no experimental validation)	Awaits animal model validation

Evidence strength definitions: see **Table 2** note.

### 6.2 Targeting the release threshold: inhibiting mtDNA leakage at the source

The release of mtDNA from mitochondria into the cytosol is the starting point of the cGAS-STING cascade. Raising the “release threshold”—i.e., reducing mtDNA leakage—is a core strategy to block overactivation of this axis at its source. Based on the systematic discussion in Chapter 2, enhancing mitophagy and reducing mtDNA oxidative damage are two key approaches to raising the release threshold. This section focuses on intervention strategies with direct evidence in myocardial infarction and myocardial ischemia-reperfusion (I/R) models; these strategies are particularly applicable to the acute phase (0–3 days) after infarction, aiming to reduce mtDNA leakage at the source.

It should be emphasized that some of the strategies discussed in this section (e.g., levosimendan, PINK1/Parkin overexpression) are supported by direct evidence from myocardial infarction or ischemia-reperfusion (I/R) models. In contrast, other strategies (e.g., Nrf2 activators, mito-TEMPO, PCSK9 inhibitors, mTOR inhibitors) have been evaluated primarily in non-MI models (e.g., sepsis, diabetic cardiomyopathy, liver I/R, aging). Although mechanistic commonalities exist across these conditions, extrapolation to post-infarction remodeling should be made with caution. Where applicable, the source and strength of evidence are indicated in the text.

Approved drug. Levosimendan, in an ex vivo rat heart I/R model, regulates mitophagy through the cGAS−STING pathway and reduces infarct size; the protective effect is partially reversed by a STING agonist (40). This study directly detected STING pathway phosphorylation and represents a relatively strong line of evidence in myocardial infarction models, but it did not directly quantify mtDNA leakage, requiring further validation [Moderate evidence; direct MI model].

Gene therapy. PINK1 (PTEN−induced putative kinase 1)/Parkin overexpression is currently the best−supported strategy. Cardiac−specific PINK1 overexpression reduces mtDNA release and inhibits the cGAS−STING pathway in a pressure overload model (1); cardiomyocyte−specific Parkin overexpression similarly reduces cytosolic mtDNA accumulation and suppresses STING activation in an I/R model (12). Both studies directly measured mtDNA and STING pathway activity, which can be considered high−quality evidence [Strong evidence; direct MI/I/R models].

Preclinical small molecules/natural products. The Nrf2 (nuclear factor erythroid 2-related factor 2) activator mangiferin was shown to directly reduce mtDNA leakage and STING activation in a sepsis-induced cardiomyopathy model (101) [Moderate/indirect evidence; derived from sepsis model, requires MI validation]. The mitochondria-targeted antioxidant mito-TEMPO was shown to reduce mtDNA oxidation and inhibit STING activation in a diabetic atrial fibrillation model (4) [Moderate/indirect evidence; derived from diabetic model, requires MI validation]. Although the evidence for these two strategies comes from sepsis and diabetic models, respectively, oxidative stress is a common pathological mechanism in various cardiovascular diseases; therefore, these strategies have potential value for cardioprotection in myocardial infarction, but their direct evidence in pure myocardial infarction models awaits validation.

In summary, enhancing mitophagy (especially the PINK1/Parkin pathway) currently appears to be the best supported strategy for raising the release threshold. Levosimendan is already approved and has a good safety profile; clinical studies in post-infarction remodeling are recommended as a priority. Nrf2 activators and mito-TEMPO are potential candidates, but they need more direct validation in myocardial infarction models. The above strategies are particularly suitable for acute-phase intervention to interrupt the initiation of positive feedback loops. (For clinical translation challenges, see Section 6.1.)

### 6.3 Targeting the activation threshold: blocking STING signaling in recipient cells

As described in Chapter 3, excessive STING activation is a key driver of post-infarction remodeling. Therefore, raising the “activation threshold”—i.e., inhibiting overactivation of the STING pathway in recipient cells—is a core strategy. This section focuses on interventions with direct evidence in myocardial infarction and myocardial ischemia-reperfusion (I/R) models; these strategies are particularly applicable to the chronic phase (>7 days) after infarction, aiming to block STING-mediated persistent inflammation and fibrosis.

Small molecule STING inhibitor. H-151 is a selective STING inhibitor (covalently binds to the Cys91 site, blocking palmitoylation). In myocardial infarction and I/R models, H-151 inhibits the cGAS-STING pathway, reduces cardiomyocyte apoptosis and fibrosis, and improves cardiac function (51, 102). It also reverses adverse cardiac outcomes in a TET2 (ten-eleven translocation 2) deficiency-associated clonal hematopoiesis model (103). This strategy has been supported by multiple independent teams, providing relatively strong evidence [Moderate evidence; multi-team validation in MI/I/R models].

Natural products. Curcumol directly binds to the STING protein and blocks its interaction with TBK1 (TANK-binding kinase 1). In a mouse model of myocardial infarction, oral administration of curcumol increases survival and attenuates ventricular remodeling; the effect is abolished in STING knockout mice, confirming its on-target action (104). PTS (Pancratium triflorum saponin, also known as notoginseng triterpene saponin) directly binds STING, blocks STING signaling in macrophages, and reduces inflammation and reverses remodeling in a rat model of acute myocardial infarction (39).Promoting STING degradation. USP20 (ubiquitin-specific protease 20) promotes autophagic degradation of STING by deubiquitinating p62, alleviating inflammation in a diabetic cardiomyopathy model (45); its efficacy in pure myocardial infarction remains to be validated. 9-PAHSA (9-palmitic acid-hydroxystearic acid) promotes STING degradation via the LKB1 (liver kinase B1)/AMPK (AMP-activated protein kinase)/ULK1 (unc-51 like autophagy activating kinase 1) pathway and has been directly shown to protect coronary microvessels in a cardiac I/R model (105) [Moderate/indirect evidence; requires MI validation]. These strategies are still at an early exploratory stage, but promoting degradation may partially preserve basal STING immune functions, representing a more promising direction.

Cell-type-specific interventions. Genetic evidence has confirmed that macrophage- or cardiomyocyte-specific STING knockout attenuates remodeling (see Sections 3.2 and 3.3), providing a theoretical basis for developing cell-targeted delivery systems (e.g., nanoparticles or AAV).

In summary, among strategies targeting the activation threshold, H-151 appears to have strongest evidence in myocardial infarction models, and natural products such as curcumol and PTS also show protective effects. Promoting STING degradation may represent a more promising direction, but all are still at the preclinical stage and urgently need clinical trial validation. The above strategies are particularly suitable for chronic-phase intervention to interrupt the sustained amplification of positive feedback loops. (For clinical translation challenges, see Section 6.1.)

### 6.4 Time window and combination targeting strategies

The previous sections described targeting strategies for raising the release threshold and the activation threshold, respectively. As outlined in Chapter 5, post-infarction remodeling can be divided into an acute phase (0–3 days, inflammation-dominated), a subacute phase (3–7 days, fibrosis initiation), and a chronic phase (>7 days, sustained remodeling). This division is based on the temporal dynamics of STING activation and the dominant timing of each pathological effect. Time-window-based combination strategies hold promise for precision intervention, as shown in **Table 4**.

Based on the above time-window characteristics, stage-specific intervention directions can be designed. In the acute phase, the focus is on raising the release threshold; representative strategies include levosimendan, mito-TEMPO, and Nrf2 activators (see Section 6.2). In the subacute phase, the focus is on raising the transmission threshold; although no clinically available strategy exists yet, proof-of-concept studies have shown that cardiac-specific downregulation of Ambra1 inhibits Ambra1^+^ sEV release and attenuates fibrosis (5). In the chronic phase, the focus is on raising the activation threshold; STING inhibitors such as H-151 or natural products like curcumol can be used (see Section 6.3).

Because the three thresholds are coupled by positive feedback, targeting multiple thresholds simultaneously may be more effective than single−agent therapy. For example, combining mito−TEMPO (reducing mtDNA oxidative leakage) with H−151 (inhibiting STING) could synergistically suppress the cGAS−STING axis at both the source and signal amplification steps, potentially interrupting the vicious cycle described in Section 5.5. However, this hypothesis has not yet been experimentally validated and urgently requires evaluation in animal models [Hypothesis; no experimental validation]. (For clinical translation challenges, see Section 6.1.)

The conceptual framework presented in this review has several important limitations that should be considered when interpreting the model:

Lack of quantitative definitions: The exact numerical values or concentration ranges for the release, transmission, and activation thresholds have not been experimentally established. The three−state classification (physiological homeostasis, compensation, decompensation) remains speculative.

Unknown endosomal escape machinery in the transmission threshold: While endosomal escape is postulated as a critical determinant of the transmission threshold, the specific molecular mechanisms that allow mtDNA to escape from endosomes/lysosomes into the cytosol are completely unknown.

Limited functional validation of cell−type−specific activation thresholds: The reported differences in activation thresholds among macrophages, cardiomyocytes, endothelial cells, and fibroblasts are largely inferred from transcriptomic data and indirect functional readouts. Direct dose−response measurements of STING sensitivity in primary cardiac cells are lacking.

Extrapolation of therapeutic evidence: Several preclinical strategies discussed in Section 6 (e.g., Nrf2 activators, mito−TEMPO, PCSK9 inhibitors) have been tested predominantly in non−myocardial infarction models (e.g., sepsis, diabetes, liver I/R). Their efficacy and safety in post−infarction remodeling remain to be directly validated.

No systematic perturbation experiments: The predicted interdependencies among the three thresholds (e.g., compensatory changes when one threshold is genetically or pharmacologically manipulated) have not been experimentally tested. Direct causal evidence linking threshold modulation to disease outcomes is still missing.

These limitations underscore that the three−threshold model is a hypothesis−generating synthesis intended to guide future research, rather than an established paradigm.

## 7 Conclusions and perspectives

### 7.1 Core conclusions

Based on the systematic discussion in the previous six chapters, this review systematically proposes a conceptual “three-threshold model” as a framework to understand post-infarction ventricular remodeling. While the model is supported by several lines of indirect and preliminary evidence, it should be viewed as a hypothesis-generating synthesis rather than an established paradigm. Direct experimental validation of each threshold and their interactions is urgently needed. This model identifies overactivation of the mtDNA-cGAS-STING axis as a core driving mechanism of post-infarction remodeling, with the degree of activation controlled by three sequential and interconnected thresholds: the release threshold (the critical point at which mtDNA escapes from mitochondria into the cytosol, Chapter 2), the transmission threshold (the efficiency with which mtDNA is transported from donor to recipient cells, Chapter 4), and the activation threshold (the sensitivity of the STING response in recipient cells, Chapter 3). The release threshold depends on mitophagy efficiency, the degree of mtDNA oxidation, and the mitochondrial damage load. The transmission threshold is determined by the form of the signal carrier and the uptake efficiency of recipient cells. The activation threshold exhibits marked cell type specificity, being lowest in macrophages and highest in fibroblasts.

Four pathological processes (inflammation, fibrosis, electrical remodeling, and vascular dysfunction) continuously lower the three thresholds through positive feedback loops, driving irreversible remodeling. Inflammatory cytokines damage mitochondria and lower the release threshold; hypoxia promotes vesicle release and lowers the transmission threshold; inflammatory cytokines and calcium overload lower the activation and release thresholds, respectively; and upregulation of IFI16 lowers the activation threshold. The three thresholds are mutually coupled; once they are persistently lowered beyond a critical point, remodeling enters a self-accelerating, irreversible phase. Time-dependent combination strategies based on the three-threshold model provide a new paradigm for precision intervention: acute phase (0–3 days) focusing on raising the release threshold (levosimendan, mito-TEMPO); subacute phase (3–7 days) focusing on raising the transmission threshold (currently only proof-of-concept, e.g., targeting Ambra1); chronic phase (>7 days) focusing on raising the activation threshold (H-151, curcumol). Combination interventions (e.g., mito-TEMPO + H-151) are expected to synergistically interrupt the positive feedback loops. This shift—from intracellular to intercellular, from single molecules to network nodes, from descriptive mechanisms to a threshold model—is reshaping our understanding of post-infarction ventricular remodeling.

### 7.2 Future directions

Clinical translation and evidence limitations. A majority of the therapeutic strategies targeting the three thresholds, particularly those aimed at the transmission threshold, are currently supported by preclinical evidence from non-myocardial infarction models or proof−of−concept studies. Direct validation of their efficacy and safety in post−infarction remodeling is urgently needed. This limitation should be considered when interpreting the translational potential of the three−threshold model.

This review has identified several key knowledge gaps. Research recommendations are categorized by threshold, with an emphasis on experimentally testable definitions for each threshold.

Release threshold: Live−cell imaging systems should be established to track mtDNA release dynamics in real time (e.g., using mitochondrial−targeted GFP or mtDNA−binding fluorophores). Mathematical models integrating mitophagy efficiency, oxidative damage load, and mitochondrial injury should be constructed (e.g., multiparameter logistic regression or machine learning models based on *in vitro* and *in vivo* data) to predict changes in the release threshold under different pathological conditions. Multi−morbidity animal models are needed to dissect how aging and diabetes synergistically lower the release threshold. Dynamic monitoring techniques for autophagic flux should be developed to screen specific enhancers of autophagic flux. In addition, in non−myocardial infarction models (e.g., hepatic I/R, aging, fatty liver), several strategies (such as PCSK9 inhibitors, mTOR inhibitors, and geniposide) have shown potential to modulate the release threshold, but direct evidence in myocardial infarction remains to be validated (15, 28, 103).

Activation threshold: Systematic dose−response studies should be performed to quantify the minimal mtDNA concentration required to activate the cGAS−STING pathway in each cardiac cell type (macrophages, cardiomyocytes, endothelial cells, fibroblasts). This can be achieved by titrating purified mtDNA or mtDNA−loaded vesicles into primary cell cultures and measuring STING phosphorylation, IRF3/NF−κB activation, and downstream cytokine production. Such experiments would provide the first quantitative definition of the activation threshold. Given the coexistence of mitochondrial and nuclear DNA in the infarct microenvironment, comparative studies using purified nuclear DNA as a stimulus are also needed to dissect the relative contributions of these two DNA sources to cGAS−STING activation in different cell types. Single−cell RNA−seq and spatial transcriptomics should be used to resolve cellular heterogeneity in the infarct zone, and cell−type−specific STING reporter mice should be generated. Small−molecule activators of USP20 should be screened, and the cardioprotective effects of 9−PAHSA should be validated. The efficacy of STING inhibitors should be tested in comorbidity models of myocardial infarction. IFI16 knockout mice should be generated to dissect the functional division between IFI16 and cGAS.

Transmission threshold: Quantitative assays for endosomal escape efficiency and vesicle uptake rates should be developed using fluorescently labeled mtDNA or pH−sensitive reporters (e.g., dual−labeled vesicles with pH−sensitive and pH−insensitive fluorophores). The fraction of internalized mtDNA that reaches the cytosol can be calculated by comparing cytosolic mtDNA levels with total cell−associated mtDNA. These measurements would yield an operational definition of the transmission threshold. CRISPR screens are urgently needed to identify specific phagocytic receptors for mtDNA in macrophages. The biogenesis mechanisms of Ambra1^+^ sEVs should be elucidated. The direct effect of fibroblast−derived mt−sEVs on the STING pathway should be validated, and the synergy between NLRP3 and cGAS−STING should be investigated. High−throughput screening based on the structure of Ambra1 to develop small−molecule inhibitors that specifically block the release or uptake of Ambra1^+^ sEVs represents a potential drug discovery direction.

Clinical translation: The axis and its intercellular communication network should be validated in human myocardial infarction samples. Prospective cohort studies should evaluate circulating mtDNA, MitoEVs, and SIRT6 as predictive biomarkers for remodeling risk. The synergistic effect of combination interventions (e.g., mito-TEMPO + H-151) should be validated. Companion diagnostic markers based on threshold phenotyping should be developed. Furthermore, research should be advanced at four levels: technology (live-cell imaging, spatial transcriptomics, single-cell multi-omics), mechanisms (phagocytic receptors, vesicle biogenesis, STING-NLRP3 synergy), translation (cell-type-specific STING inhibitors, MitoEV liquid biopsy, companion diagnostics), and clinical (validation in human samples, threshold-stratified randomized controlled trials, precision subtyping), to promote the clinical translation of the three-threshold model.
