# Supplementary material for: Mitochondrial DNA-driven intercellular communication networks in post-infarction ventricular remodeling: the three-threshold model of cGAS-STING activation
Source: Front Immunol. 2026 May 25;17:1852079. doi: 10.3389/fimmu.2026.1852079 (PMC13243067; doi:10.3389/fimmu.2026.1852079)
Supplement: Supplementary file 1 [file Table1.docx]

**Supplementary Material**

**Supplementary Table S1. Evidence strength, key studies, and experimental models for core findings**

| **Core finding** | **Key supporting studies** | **Experimental model(s)** | **Evidence strength** |
| --- | --- | --- | --- |
| PINK1/Parkin deficiency → mtDNA leakage → cGAS‑STING activation | Zhou H et al., 2025 (Cardiovasc Res); Zhou G et al., 2024 (Genes Dis); Li Y et al., 2026 (Basic Res Cardiol, in press) | Pressure overload, MI/I/R models; multiple independent teams | Strong |
| cGAS‑STING pathway activation in post‑MI remodeling | Shi F et al., 2025 (J Clin Biochem Nutr); Zhou G et al., 2024 (Genes Dis); Guo Y et al., 2023 (Theranostics); Meng S et al., 2026 (Theranostics); Yao H et al., 2025 (Acta Pharm Sin B) | MI, I/R, pressure overload models; human hypertensive hearts; global and cell‑specific knockouts | Strong |
| Mode 1: Cardiomyocyte → Macrophage (naked mtDNA) | Meng S et al., 2026 (Theranostics) | Diabetic atrial fibrillation model (single study; awaits MI validation) | Moderate |
| Mode 2: Cardiomyocyte → Fibroblast (Ambra1⁺ sEVs) | Zhang C et al., 2023 (Sci Bull) | MI/R model (single study; awaits independent validation) | Moderate |
| Mode 3: Fibroblast → Macrophage (mt‑sEVs) | Zhao Y et al., 2025 (Pharmacol Res); Hu Y et al., 2024 (Eur Heart J, conference abstract) | MI model (single study + conference abstract; cGAS‑STING dependence unclear) | Weak/Preliminary |
| Mode 4: Injured cell → Endothelial cell (intact mitochondria) | Li S et al., 2022 (Am J Transplant) | Transplant rejection model; ex vivo heart perfusion (direct cardiac evidence limited) | Moderate |
| Levosimendan (release threshold) | Shi F et al., 2025 (J Clin Biochem Nutr) | Ex vivo rat heart I/R model | Moderate |
| PINK1/Parkin overexpression (release threshold) | Zhou H et al., 2025 (Cardiovasc Res); Li Y et al., 2026 (Basic Res Cardiol, in press) | Pressure overload, I/R models | Strong |
| Nrf2 activators (mangiferin) | Song J et al., 2026 (Chin Med) | Sepsis‑induced cardiomyopathy model (requires MI validation) | Moderate/indirect |
| mito‑TEMPO | Meng S et al., 2026 (Theranostics) | Diabetic atrial fibrillation model (requires MI validation) | Moderate/indirect |
| H‑151 (STING inhibitor) | Hu S et al., 2022 (Int Immunopharmacol); Wang X et al., 2025 (Signal Transduct Target Ther); Dou Y et al., 2024 (Clin Transl Med) | MI, I/R models; multiple independent teams | Moderate |
| Curcumol, PTS | Yang N et al., 2025 (Phytomedicine); Yao H et al., 2025 (Acta Pharm Sin B) | MI models (single studies) | Moderate |

Table note: Evidence strength definitions as in Tables 1, 2 and 4. This table is provided as supplementary material to enhance transparency and readability. For detailed discussion, see corresponding sections in the main text.
